# How a concerned family member, friend or member of the public can help someone with gambling problems: a Delphi consensus study

**DOI:** 10.1186/s40359-016-0110-y

**Published:** 2016-02-03

**Authors:** Kathy S. Bond, Anthony F. Jorm, Helen E. Miller, Simone N. Rodda, Nicola J. Reavley, Claire M. Kelly, Betty A. Kitchener

**Affiliations:** Mental Health First Aid Australia, Level 6/369 Royal Parade, Parkville, VIC 3052 Australia; Centre for Mental Health, Melbourne School of Population and Global Health, The University of Melbourne, Level 4/207 Bouverie Sreet, Parkville, VIC 3010 Australia; Victorian Responsible Gambling Foundation, Level 6, 14-20 Blackwood Street, North Melbourne, VIC 3051 Australia; Turning Point, Eastern Health, 54-62 Gertrude Street, Fitzroy, VIC 3065 Australia; School of Public Health and Psychosocial Studies, AUT University, 90 Akoranga Drive, Auckland, 1142 New Zealand; School of Psychology, Deakin University, 1 Gheringhap Street, Geelong, VIC 3220 Australia

**Keywords:** Gambling problems, Consumers, Caregivers, Significant others, Mental health first aid, Signs of gambling problems

## Abstract

**Background:**

Gambling is an enjoyable recreational pursuit for many people. However, for some it can lead to significant harms. The Delphi expert consensus method was used to develop guidelines for how a concerned family member, friend or member of the public can recognise the signs of gambling problems and support a person to change their gambling.

**Methods:**

A systematic review of websites, books and journal articles was conducted to develop a questionnaire containing items about the knowledge, skills and actions needed for supporting a person with gambling problems. These items were rated over three rounds by two international expert panels comprising people with a lived experience of gambling problems and professionals who treat people with gambling problems or research gambling problems.

**Results:**

A total of 66 experts (34 with lived experience and 32 professionals) rated 412 helping statements according to whether they thought the statements should be included in these guidelines. There were 234 helping statements that were endorsed by at least 80 % of members of both of the expert panels. These endorsed statements were used to develop the guidelines.

**Conclusion:**

Two groups of experts were able to reach substantial consensus on how someone can recognise the signs of gambling problems and support a person to change.

**Electronic supplementary material:**

The online version of this article (doi:10.1186/s40359-016-0110-y) contains supplementary material, which is available to authorized users.

## Background

Gambling is an enjoyable recreational pursuit for many people. However, for some it can lead to significant problems for the individual and their family, such as financial and legal problems, psychological distress, and relationship and family stress [1–4]. Gambling problems are often defined as gambling activities where the person struggles to limit the amount of money or time spent on gambling [5]. However, these defining characteristics are not overt, potentially making gambling problems hidden from family members, friends and co-workers of the gambler. While the signs of gambling problems remain largely unrecognised, there is limited possibility of support and encouragement from others.

### Warning signs of gambling problems

There is limited research investigating the signs of gambling. One exception is the recent research to develop and evaluate the Gambling Behaviour Checklist (GBC) that is used by gambling venue staff [6–9]. The GBC is a validated list of observable signs of gambling problems shown in gambling venues. The signs of problem gambling as described by the GBC [9] includes losing control over gambling, (e.g. difficulty stopping at closing time), seeking funds to gamble (e.g. withdraws cash from bank account multiple times), gambling intensely (e.g. fast play) or for a long duration (more than 3 h), displaying superstitious behaviour, having an emotional response to losing, and displaying unusual social behaviour (e.g. avoids contact or conversations with others). The use of the GBC was shown to encourage staff follow-up actions with identified customers, usually in the form of an informal chat with the customer.

These signs may be evident to some family, friends or co-workers of a person with gambling problems, if they go to gambling venues with the person. However, research indicates that many people are not aware of the extent of the gambling problems or even that the person is gambling at all [10]. Evans and Delfabbro [11] and Hing, Nuske and Gainsbury [12] have recommended that community training includes teaching family, friends and co-workers to recognise the signs that may indicate a person has gambling problems and how to support and give advice to a person with gambling problems.

### Family support

When family is aware of gambling problems, they can be important to recovery. One intervention for family members is Community Reinforcement and Family Training (CRAFT). CRAFT provides skills training to family members for coping with a loved one’s gambling problems. This intervention has been shown to reduce the frequency or amount of time spent gambling and the negative consequences of gambling [13–16]. While this family intervention appears to be helpful in gambling recovery, it is dependent on family members recognising the gambling problems and seeking professional help.

Another way that family (and others) can encourage recovery from gambling problems is to provide reliable information about gambling and encourage help-seeking. To our knowledge, there is no research to suggest that this is helpful for gambling problems, even though this approach has been found to be effective across other mental health problems. For example, research shows that the provision of mental health information to a person increases the likelihood that they will seek help and adhere to treatment, and improves the prognosis and self-management of mental health problems [17, 18]. It is likely that information from family, friends or co-workers about gambling problems will encourage help-seeking, support recovery and reduce gambling harms.

### Gambling harms

Gambling can cause significant problems for the individual, their family and the community. Problem gamblers self-report poorer health, psychological distress, smoking and alcohol abuse [1]. Furthermore, suicidal thoughts and behaviours are more common amongst problem gamblers and their children [19]. Partners of problem gamblers also report significant harms, including relationship conflict, financial problems and poorer self-reported health [20, 21], and that their children’s emotional and physical health has been negatively affected by problem gambling [21, 22]. There is also a strong link between gambling problems and other mental health problems, with an international systematic literature review finding that problem gamblers had high rates of substance use disorders (58 %), mood disorders (38 %) and anxiety disorders (37 %) [23]. Given these significant harms, support and encouragement from family members, friends and co-workers to seek help for gambling problems is important.

### Seeking help for gambling problems

Few people with gambling problems seek professional treatment, with the likelihood increasing with the severity of the problems. Slutske [24] found that, of those who experienced five symptoms of pathological gambling (according to the DSM-IV), only 4 % sought professional help. This percentage increased with the number of symptoms to 6, 17, 31 and 76 % of people with 7, 8, 9 and 10 symptoms, respectively. Another study found that professional help-seeking tended to occur only after the experience of significant harms from gambling [25].

Research by Hing et al. [12] has identified the motivators and barriers to help-seeking (both professional and informal help-seeking). The strongest motivators for help-seeking involve financial, relational and emotional harms associated with gambling, e.g. relationship problems, problems at work, problems with housing and legal problems. Professional help-seeking usually follows a significant crisis, and is often preceded and followed by informal help-seeking. One of the stronger motivators for seeking treatment identified in this study was “pressure from family or friends”. However, research indicates that very few people receive encouragement to seek help for their gambling problems from friends and family, with problem gamblers being more likely to receive this encouragement than moderately at risk gamblers [26]. Another identified motivator was “concern from the venue where [the person] was gambling”, although this was a less strong motivator than “pressure from family and friends” [12]. This finding may indicate that while using a venue checklist will help some people with gambling problems, educating family and friends to recognise the warning signs and provide support may be more effective in recovery.

The barriers to help-seeking identified in the literature are: a desire of the person to handle problems on their own; shame, embarrassment and stigma; an unwillingness or inability to admit that there is a problem; or minimisation of the problems associated with gambling [12, 24, 27, 28]. If family members, friends and co-workers can non-judgementally support a person to recognise and admit significant problems associated with their gambling, the person may be more motivated to seek help and recover.

### Training for family members, friends and co-workers

Two potential forms of training for family, friends and co-workers to encourage help-seeking are the provision of guidelines for how to help a person with gambling problems and training courses. Guidelines, using the Delphi method, have been developed on how members of the public can recognise and assist a person who has a mental health problem or is in a mental health crisis situation (e.g. they are suicidal), including guidelines for depression [29], psychosis [30], problem drinking [31], problem drug use [32], eating disorders [33], suicidal thoughts and behaviours [34], non-suicidal self-injury [35], panic attacks [36] and traumatic events [37].

Guidelines in themselves may not ensure change in supportive behaviours. Therefore, these guidelines have been used to inform the contents of the Mental Health First Aid (MHFA) course [38, 39]. People who receive MHFA training have greater knowledge regarding mental health, less negative attitudes and show increased supportive behaviours toward individuals with mental health problems [40].

Given the significant harms associated with and the hidden nature of gambling problems, and the importance of family support in recovery, this study aimed to develop mental health first aid guidelines on how to help a person with gambling problems. Specifically, we aimed to: (1) determine, using the Delphi research method, how members of the community can best help a person who has gambling problems; (2) develop a list of evidence-informed, observable signs that a member of the public can use to help identify a person who may have gambling problems; and (3) produce a guidelines document that is available to the public and that will inform Mental Health First Aid training based on the findings of the Delphi research project.

## Methods

The Delphi process [41] is an expert consensus method that can be used to develop best practice guidelines using practice-based evidence. An advantage of the Delphi method is that the expert opinion is gathered anonymously through the use of online (or postal) surveys, allowing for all participants on the panel to equally influence the results. Development of the current guidelines involved four steps: (1) formation of the expert panels, (2) literature search and survey questionnaire development, (3) data collection and analysis, and (4) guidelines development.

### Step 1: Panel formation

As described by Hasson, Keeney and McKenna [42], the Delphi method usually involves the use of one expert panel, often professionals working in the area of study. However, more recent work in the mental health field has included multiple panels, including consumer and carer experts, allowing for lived experience expertise to influence guidelines development (e.g., [43]). This current study utilised two expert panels: (1) mental health professionals with experience working with people with gambling problems and gambling researchers (professional panel), and (2) people with personal experience of gambling problems in themselves or others close to them (lived experience panel). See Table 1 for the inclusion criteria. The aim was to recruit a minimum of 30 people to each panel, which is within the typical Delphi panel size of 15–60 experts [42], allowing for reliable consensus to be reached.

**Table 1:** Inclusion criteria

Panel	Criteria
Professional	• Be 18 years or older, AND • Live in Australia, Canada, Ireland, New Zealand, United Kingdom or the United States, AND • Have a minimum of 2 years’ experience specialising in research on or treatment of problem gambling.
Lived experience	• Be 18 years or older, AND • Live in Australia, Canada, Ireland, New Zealand, United Kingdom or the United States, AND • Have a lived experience of gambling problems, but are currently recovered and have experience in an advocacy or peer support role, OR • Are a family member or friend who has assisted a person with a gambling problem and have experience in an advocacy or peer support role.

### Step 2: Literature search and survey questionnaire development

**Fig. 1:**
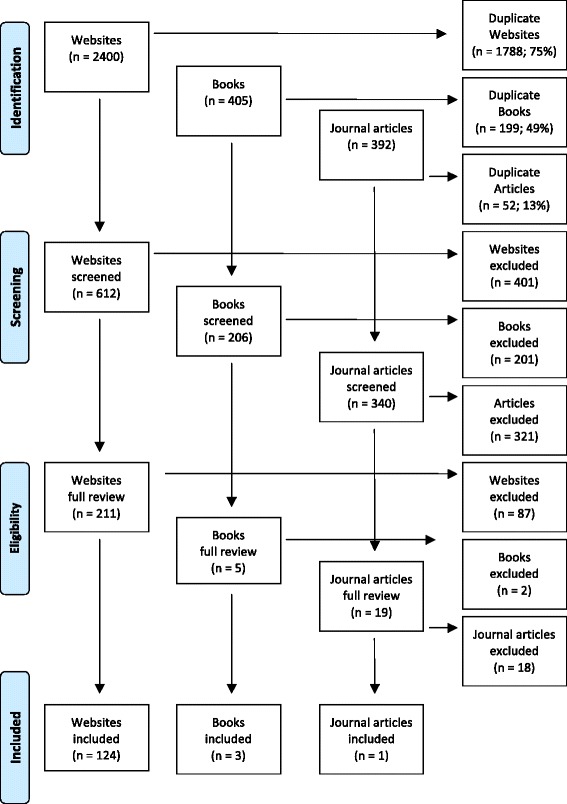
Results of literature review

In order to inform the content of the initial questionnaire sent out to the expert panel members, a systematic search of the ‘grey’ and academic literature was conducted in July 2014 to gather statements about how to help someone with gambling problems. The search was conducted using Google Australia, Google UK, Google USA, Google Books and Google Scholar. The key search terms used were: (problem gambling), (pathological gambling), (gambling addiction), (compulsive gambling), (gambling AND mental health), (gambling AND mental illness), (helping someone who gambles), (help a friend stop gambling), (treatment for gambling), (help for gambling), (guide for problem gambling), (problem gambling harm), (Gam-anon), (gambling spouse), (gambling partner), and (living with a gambler). The first 50 websites, books and journal articles were retrieved and duplicates were excluded. The remaining sources were reviewed for relevant information. Any links appearing on the websites were also reviewed. Websites, articles and books were excluded if they did not contain information about how a member of the public can recognise and help a friend or family member who has gambling problems. A total of 128 resources were included and used to develop the Round 1 survey. These resources included websites developed by (1) government sponsored, non-profit and private gambling help organisations/treatment centres; (2) gambling research centres; and (3) on-line support services for family of people with gambling problems. Figure 1 summarises the results of the literature search.

A working group, consisting of staff from Mental Health First Aid Australia, the University of Melbourne, Turning Point and the Victorian Responsible Gambling Foundation translated the relevant information from the literature search into helping statements that were clear, actionable, and contained only one idea. The statements were used to form a questionnaire, involving three survey rounds, that was administered to the expert panels via SurveyMonkey. The panel members were asked to rate each of the statements, using a 5-point scale (‘essential’, ‘important’, ‘don’t know/depends’, ‘unimportant’ or ‘should not be included’), according to whether or not they thought the statement should be included in the guidelines. See Additional file 1 for copies of the 3 rounds of the questionnaire.

In this study we made a distinction between *problem gambling* (a diagnosis) and the subclinical symptoms of problem gambling. We use the term *gambling problems* defined as gambling activities where the person struggles to limit the amount of money or time spent on gambling, which leads to adverse consequences for the person, their friends and family, or for the community. This could include someone whose gambling problems are at a clinically diagnosable level [5]. This definition was used because it is not feasible or preferred that family members diagnose disordered or pathological gambling, and because the study sought to identify the signs of a range of problems (from at risk gambling through to problem gambling). Also if family, friends and co-workers can identify and address the signs earlier, severe gambling harms may be prevented.

### Step 3: Data collection and analysis

Data were collected in three survey rounds administered between January and April 2015. In Round 1, panel members also had the opportunity to provide qualitative data in the form of comments or suggestions for new helping statements. The qualitative data were collected by asking, “Are there any additional statements you think are important to giving help to a person with gambling problems? Please write your suggestions in the box provided.”

After panel members completed a survey round, the statements were categorised as follows:

1. Endorsed. The item received an ‘essential’ or ‘important’ rating from 80–100 % of members of both panels.
2. Re-rate. The item received an ‘essential’ or ‘important’ rating from 70–79 % of members from both panels, or an ‘essential’ or ‘important’ rating from 70–79 % of members from at least one panel and above 80 % from the other panel.
3. Rejected. The item did not fall into either the endorsed or re-rate categories.

The participants’ comments were thematically analysed by the working group. The working group used the following criteria to determine whether the participants’ comments would be translated into new helping statements: (1) the idea was understandable and actionable, (2) it was not included in the first survey, and (3) it was within the scope of the project. This new content was translated into clear and actionable statements for the Round 2 survey. The Round 2 survey also included the items from Round 1 to be re-rated. Panel members were given a summary report of Round 1 that included a list of the items that were endorsed and rejected, as well as the items that were to be re-rated in the next round. The report included the panel percentages of each rating, as well as the panel member’s individual scores for each item to be re-rated. This allowed the participants to compare their ratings with each expert panel’s consensus rating and consider whether to maintain or change their answer when re-rating an item.

The procedures for Rounds 2 and 3 were the same as described above with several exceptions. Round 2 consisted of new items from the Round 1 comments, there was no opportunity for comments in Round 2 or Round 3, and if a re-rated item did not receive an ‘essential’ or ‘important’ rating by 80 % or more of each panel, it was rejected. Round 3 consisted of any new items included in Round 2 that needed to be re-rated, according to the above criteria.

### Step 4: Guidelines development

All of the endorsed statements were written into prose to form the guidelines document. The first author drafted the guidelines by grouping the list of endorsed statements into sections based on common themes. Where possible, statements were combined and repetition deleted. The working group edited the draft to produce the final guidelines document. This document was given to the expert panel members for comment and final endorsement.

### Ethics

This research was approved by the University of Melbourne Human Ethics Committee. Informed consent was obtained from all participants by clicking ‘yes’ to a question about informed consent in the Round 1 survey.

## Results

Initially we had hoped to form three expert panels – professionals, people with a history of gambling problems, and family or friends of a person with gambling problems (affected others). However, despite an extensive search, it was difficult to recruit enough ‘affected others’ panel members to yield stable results. A panel size of 23 has been found to yield stable results in a simulation study [44]. As a result of the small number of people recruited to the ‘affected others’ panel and because the item endorsement rates in the Round 1 questionnaire were highly correlated between the ‘affected others’ and ‘people with a history of gambling problems’ panels (r = 0.80), the two panels were combined into one ‘lived experience’ panel.

A total number of 66 people were recruited, 34 to the ‘lived experience’ panel (6 ‘affected others’ and 28 people with a history of gambling problems) and 32 to the ‘professional panel’. The retention rate for completing all three rounds was 69.5 % (see Table 2 for the breakdown of the retention rate for each of the panels). Participants who completed all three rounds were 42.4 % male and 57.6 % female, and had an average age of 49.9 years (12.6 SD, range 23–73). Participants were from Australia (60 %), North America (21 %), New Zealand (17 %) and the UK (3 %).

**Table 2:** Retention rates from Round 1 to Round 3

Expert panel		Invited	Completed Round 1	Completed Round 2	Completed Round 3	Retention rate
Lived experience		43	40	35	34	79.1 %
	HGP	36	33	29	28	77.8 %
	Affected others	7	7	6	6	85.7 %
Professionals		52	41	33	32	61.5 %
Total		95	81	68	66	69.5 %

*HGP* People with a history of gambling problems

### Endorsed items

**Fig. 2:**
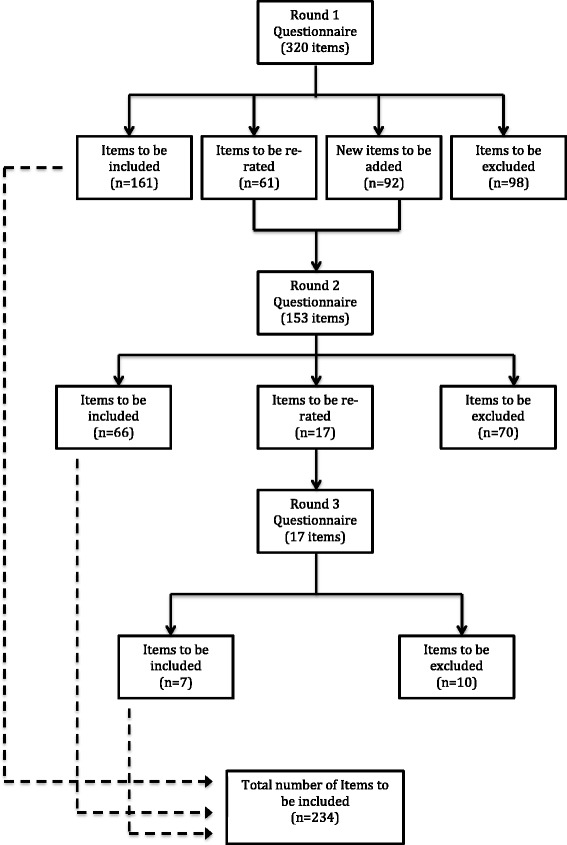
Total number of items endorsed, re-rated and rejected

A total of 412 items were rated over the three rounds to yield a total of 234 endorsed items and 178 rejected items (see Additional file 2 for a list of the endorsed and rejected items). Figure 2 presents the information about the total number of items rated, endorsed and rejected over the three rounds. The endorsed items formed the basis for the guidelines. There was a strong positive correlation between the two panels in the percentage endorsement for whether items should be included in the guidelines, (r = 0.82).

The endorsed items outlined what a family member, friend or co-worker needs to know and do to support a person with gambling problems. This includes knowing specific information about gambling and gambling problems, and the association between gambling problems and mental health problems. The guidelines also outline specific actions for approaching and talking with the person in a non-judgmental way. Furthermore, effective ways of encouraging change and help-seeking are identified, as well as how to support the person even if they do not wish to change their gambling. Strategies for managing crisis situations (e.g. suicide) are also covered. In addition, the observable signs that may be evident at home, work or in a venue were identified.

### Warning signs of gambling problems

This research developed an evidence-informed list of warning signs that a family member, friend or co-worker can use to recognise gambling problems. Seventy-seven of 153 warning signs (50.3 %) were endorsed by both panels. The list of signs that may indicate a person has gambling problems includes (see Additional file 2 for the full list of endorsed signs):

- ▪ Gambling behaviours (e.g. gambles almost every day, gambles to escape problems)
- ▪ Signs evident while gambling (e.g. stops gambling only when the venue is closing, shows significant changes in mood during a gambling session)
- ▪ Financial signs (e.g. complains about mounting debt, frequently contacted by debt collectors)
- ▪ Social signs (e.g. becomes isolated from others because of gambling, has conflicts with others about money)
- ▪ Signs evident at home (e.g. steals from family or friends to fund gambling, family members hide money from the person in order to cover living expenses)
- ▪ Signs evident in the workplace (e.g. gambles during work time, repeatedly violates workplace gambling policy).

The qualitative data suggest that recognising the signs of gambling problems may be difficult for friends, family and co-workers. For example a participant with a history of gambling problems said, “…most compulsive gamblers I know are very good at hiding most of the traits that are listed here.” and “…[my] signs [were] never noticed by my own family.” Other phrases used by panel members with a history of gambling problems to describe people with gambling problems were: “facile liars”, “deceptive and manipulative”, “good at conning folks”, and “manipulator and a liar.”

It was noted by a few of the panel members that, given the hidden nature of gambling problems, venue staff may be well suited to identify the signs of gambling problems. One participant said, “Generally a compulsive gambler will gamble secretly - often the only people who would observe the signs…are venue staff.” And another said:

“These are all important signs, but I would never display too many of them if I was at a gambling venue with family or friends. So only the gambling venue’s employees saw those things (signs of gambling problems).”

### Harm minimisation

In addition to the warning signs, a number of harm minimisation strategies were endorsed by the panel members. There were a total of 22 items that pertained to harm minimisation strategies and 16 (72.7 %) of these items were endorsed. In spite of this high level of endorsement, the qualitative data suggest strongly held negative views on harm minimisation strategies by a minority of the panel members, particularly by those who have a history of gambling problems (HGP) (see Table 3 for the comments pertaining to the harm minimisation items). Thematic analysis of the comments suggests that those who are opposed to harm minimisation strategies believe gambling problems cannot be cured, only managed through abstinence.

**Table 3:** Qualitative data about harm minimisation items

Panel	Quote
HGP	“I cannot agree with the harm minimisation as this just increases the chance of a blow out. The more you go the more you are kept in that trance and the more you need to go.”
HGP	“I believe for someone with a gambling addiction/illness it is necessary to advise them that the goal is to not gamble again, seek healthcare, seek support, be honest, develop new activities etc. As GA (Gamblers Anonymous) says don't test or tempt oneself on anything.”
HGP	“I think [this harm minimisation item] should be removed so first aider doesn’t think a CG (compulsive gambler) can become cured. I tricked both my partner and employer in thinking that I was recovered/cured and gambled for three more years and almost lost everything including my life.”
HGP	“The person should be made aware that harm minimisation does not work and will lead to a blowout. Abstinence should be encouraged.”
HGP	“This illness doesn’t allow for gambling periodically …at some times etc.....it is necessary I believe for those of us with addiction to stop entirely. Any false illusions we can gamble a little bit…with stipulations will ultimately lead to the same self destruction that brought us to our graveside chats with ourselves re suicide etc.”
HGP	“[This item about a harm minimisation strategy] is a value statement not a fact. Based on the GA…medical model of abstinence. Public Health approaches -harm minimisation and learned behaviour models do not subscribe to this view. This statement should be reviewed and changed.”
HGP	“All of these statements amount to the first aider accepting that the gamblers past actions are, to some degree, acceptable. Which can only result in further problems, in the future.”
HGP	“‘Restricting gambling activities’ is broadly accepted as being possible, only by helpers who have not been helping long enough to have seen the return of clients who have ‘busted’, whilst believing that they could become ‘social gamblers’ again.”
HGP	“All great suggestions to be followed by someone who isn’t a compulsive gambler.”
Professional	“Harm-reduction suggestions are more appropriate in the early stages of a problem.”

*HGP* People with a history of gambling problems

### Differences between groups

In spite of the strong correlations, there were also areas of disagreement. Items that were rejected by one panel but endorsed by the other, and that received notably higher or lower rating (±10 %) are noted below. Ten per cent was chosen as this was used in previous studies [43, 45].

#### Items rejected by the professional panel by + 10 %

Sixty items that were endorsed by the lived experience panel received a lower rating from the professional panel and most fell into the following categories:

- Signs that may not be evident in a professional setting (i.e. the signs seen at work, in a gambling venue or at home), for example, “The person cashes in investments or other assets early” and “The person borrows money from co-workers”.
- Items that may have been perceived as requiring the first aider to act in the role of a professional, for example items about helping the person list the advantages and disadvantages of gambling or identify problems that have led to an increase in gambling.

#### Items receiving a lower rating from the lived experience panel

There were only two items that received a lower rating from the ‘lived experience’ panel: “The first aider should be aware that the following behavioural signs indicate that a person may have gambling problems: After losing, the person uses alcohol to forget about gambling problems.” and “If the person decides to continue gambling, the first aider should encourage them to reduce the negative impact of gambling by: Keeping a record of gambling wins and losses.”

### Guidelines development

The first author grouped similar items under specific headings, re-writing them into continuous prose for ease of reading. Original wording of the items was retained as much as possible. Some items were given examples and explanatory notes to clarify the advice, for example, the risk factors for gambling problems were included in the guidelines. The working group reviewed this draft to ensure that the structure and the language were appropriate for the audience that the guidelines target. The draft guidelines were then given to panel members for final comment, feedback and endorsement. One panel member requested only minor changes related to grammar and spelling preferences.

The final guidelines (available at: www.mhfa.com.au) provide information on how to assist a person with gambling problems [46]. The main themes and subthemes, and a brief description of each section, follow:

- **What are gambling problems?** In addition to defining gambling problems, this section also touches upon the association between mental health problems and gambling problems.
- **Motivations for gambling.** This section lists the motivations for gambling and gambling problems.
- **How can I tell if someone has gambling problems?** This section includes a list of the risk factors that contribute to the development of gambling problems and the warning signs of gambling problems, grouped into the following sub-sections:
  - ▪ Gambling behaviours
  - ▪ Signs evident while gambling
  - ▪ Mental and physical health signs
  - ▪ Financial signs
  - ▪ Social signs
  - ▪ Signs evident at home (which includes signs that may be evident in family members)
  - ▪ Signs evident in the workplace
- **Approaching someone about their gambling.** This section provides communication suggestions for how to bring up and talk about gambling problems in a non-judgmental way and includes the following sub-sections:
  - ▪ How to talk to the person
  - ▪ Dealing with negative reactions
- **Encouraging professional help.** This section includes information about professional help and how to encourage a person to seek help.
- **Encouraging the person to change.** This section provides information about setting healthy boundaries with the person and practical suggestions for encouraging the person to change.
- **If the person does not want to change**. This section provides information about helping the person when they are unaware or in denial about their gambling problems.
- **Supporting the person to change.** This section includes a list of strategies that the person can use to change their gambling and includes information about supporting the person through relapse.
- **What to do if you are concerned for the safety of the person or others.** This section provides information about what to do if the person is experiencing suicidal thoughts or behaviours, or where the first aider may be concerned for the safety of others, including the person’s children or partner, or the first aider themselves.

## Discussion

This research aimed to develop a set of guidelines on how a concerned friend or family member can support a person with gambling problems. Overall, 234 items were endorsed by both expert panels as important or essential to be included in the guidelines. The endorsed items were written into a guidelines document that is available to the public.

A strength of this document is that it addresses a wide variety of topics or situations that a person may encounter when supporting someone with gambling problems. These include how to recognise the warning signs of gambling problems, how to talk to a person if you are concerned that they have gambling problems, how to encourage the person to change (including specific strategies to reduce gambling harms) and what to do if the person is resistant to changing their gambling. This final point is of particular importance, especially when considered within a reactance theory framework [47]. When talking to someone about changing their gambling behaviours, reactance, or resistance to change, may be activated when a person perceives that a freedom (in this case gambling) is threatened. When reactance is activated the person may be less motivated to change their gambling. These guidelines include information on how to support someone who may not be motivated to change their gambling.

### Warning signs and the hidden nature of gambling problems

This research identified a number of observable signs that, when several are present, indicate a person may have gambling problems. There are many lists of warning signs for gambling problems in the grey literature, some based on the DSM criteria [48] for gambling problems (e.g. is preoccupied with gambling) and others based on professional or personal experience. Our list of warning signs is evidence-informed through the use of the Delphi method and includes specific gambling behaviours; signs that are evident while gambling, at work or at home; financial signs; and signs evident in family members.

As noted earlier, only 50 % of the warning signs identified in the literature were endorsed by both panels. There is no clear reason for this low rate of endorsement. It may have been because some signs are only evident to a particular type of expert panel member (e.g. a family member), leading to low endorsement by the other panellists.

To our knowledge this is the first list developed for use by friends, family and co-workers to help identify whether someone they know is experiencing gambling problems. However, the qualitative data indicate that people with gambling problems may be skilled at hiding signs from family and friends, and that venue staff may be well placed to observe these signs. Delfabbro et al. [6, 7] and Thomas et al. [9] developed and validated the Gambling Behaviour Checklist (GBC), a list of warning signs that may be evident to gambling venue staff. The use of the GBC was shown to encourage staff follow-up actions with identified customers, usually in the form of an informal chat with the customer.

If venue staff can be trained to use a list of signs that indicate potential gambling problems, it may also be possible to train members of the public to recognise these signs and approach a person they are concerned about. Courses exist that teach people the skills needed to recognise the signs of mental health problems and give appropriate initial help, and support someone experiencing mental health problems. One such course is the MHFA course. This course is based on guidelines developed using the same process as described in this article. MHFA courses have been extensively researched and have been shown to increase the ability to recognise the signs of mental health problems, increase confidence in providing assistance to someone experiencing mental health problems, and to improve the quality of mental health first aid actions [40, 49]. It is possible that similar training based on the current set of guidelines will improve the ability of family members, friends and co-workers to recognise the signs and provide support to someone with suspected gambling problems.

Research indicates that a significant number of people who are at-risk gamblers will transition into high-risk and problem gambling over time [1]. Research also indicates that a strong motivator for help-seeking for gambling problems is “pressure” from family or friends [12]. With the help of these guidelines, family members, friends and co-workers may recognise the warning signs of gambling problems earlier, and approach the person in a supportive and non-judgmental way. With this support, the person may be motivated to seek help and may recover earlier. The flow on effect of this may be a reversal or halting of the transition into more risky gambling, and the reduction of gambling harms.

Another possible application of these guidelines is to train mental health professionals, who are not experts in gambling problems. This will help them to recognise and address gambling problems in their clients, and refer on as necessary.

### Harm minimisation strategies

A number of items were endorsed that suggest harm minimisation strategies for a person who does not want to change or abstain from gambling. Harm minimisation tends to sit within a public health model and is central to identifying and addressing gambling problems [1]. Broadly, harm minimisation strategies attempt to limit the pervasive impact of adverse health consequences associated with gambling and can target individuals and groups, the gambling environment, and public policy [50]. Our guidelines target individuals.

Harm minimisation strategies that target individuals are controversial. One argument against using harm minimisation strategies is that they might encourage people to continue the harmful behaviour [51]. This opinion was evident in the qualitative data, for example, “All of these statements (harm minimisation strategies) amount to the first aider accepting that the gambler’s past actions are, to some degree, acceptable. Which can only result in further problems in the future.”

Another way to view harm minimisation is as complementary to treatment and prevention [52]. Research further supports the notion that one does not necessarily have to abstain from gambling to recover from gambling problems. A general population study found that 90 % of the participants who recovered from their gambling problems did so without abstaining fully from gambling [53]. That 73 % of the harm minimisation items were endorsed supports the notion that there is a place for harm minimisation strategies in gambling recovery.

### Limitations

There are a few limitations to this study that are worth mentioning. First, there is limited research that indicates what is most helpful for people with gambling problems, therefore, the initial literature search may not have identified all relevant strategies. Another limitation is the possibility that some panel members were asked to advise on statements that were beyond their expertise, possibly leading to a lack of inclusion of useful items. Furthermore, while participants are able to provide comments in Round 1 of the survey, they are not able to discuss their comments and opinions with others. Panel members may have held biases or made incorrect assumptions that were unchallenged because there was no opportunity for discussion. It is possible that key actions were omitted from the guidelines because of this.

The scope of this project was limited to helping statements that centred on the person with gambling problems, excluding helping actions for affected others. Family members are impacted by a person’s gambling problems and often require professional assistance for problems related to their loved one’s gambling [54]. Unfortunately, including items related to supporting affected others would have made the survey too lengthy and put undue burden on the participants. Finally, these guidelines were developed for English-speaking Western countries and further research is need to adapt them for other cultures.

Future research to develop guidelines for helping affected others would be beneficial. It would also be helpful to use the Delphi method to develop guidelines for specific groups of people, such as Indigenous Australians, people from culturally and linguistically diverse backgrounds, and adolescents. Other research could validate the various signs identified in this study, or evaluate downloads of these guidelines from the Web, as has been done with previous guidelines [45]. Finally, any training that is developed using these guidelines should be evaluated.

## Conclusion

Given the significant harms that result from gambling problems, these guidelines will provide needed guidance on how a concerned friend, family member or co-worker can recognise and support someone with gambling problems. People with a lived experience of gambling problems and professionals who treat people with gambling problems were able to reach consensus about a number of strategies for assisting a person with gambling problems. It is anticipated that these guidelines will inform future training and will be used by individuals to support people with gambling problems.

## Additional files

Additional file 1:
**Survey Rounds 1, 2 and 3.** (PDF 599 kb) Additional file 2:
**Endorsed and Rejected items.** (XLSX 48 kb)

## Abbreviations

GBC: Gambling Behaviour Checklist
HGP: people with a history of gambling problems
MHFA: mental health first aid
