# Supplementary material for: How a concerned family member, friend or member of the public can help someone with gambling problems: a Delphi consensus study
Source: BMC Psychol. 2016 Feb 3;4:6. doi: 10.1186/s40359-016-0110-y (PMC4739356; doi:10.1186/s40359-016-0110-y)
Supplement: Additional file 1: — Survey Rounds 1, 2 and 3. (PDF 599 kb) [file 40359_2016_110_MOESM1_ESM.pdf]

Helping a person with gambling problems

Information about this survey

Purpose of the research

The purpose of this project is to develop a set of guidelines for the public on how to support someone with gambling problems to seek help and recover.

Your role

You have been selected as a panel member for this study because you are 18 years or over and:

• Have a **lived experience of gambling problems**, but are currently recovered and have experience in an advocacy or peer support role,

OR

• Are a **family member or friend** who has assisted a person with a gambling problem and have experience in an advocacy or peer support role,

OR

• Have a minimum of 2 years' experience **specialising in research on or treatment of** problem gambling.

Your task is to rate the statements presented in this questionnaire according to how important you believe they are for providing guidance to adults helping a person with gambling problems to seek help and recover.

How this questionnaire was developed

The statements in this questionnaire were derived from information collected during a literature review of websites, books, and journal articles. This review examined any written information about how a member of the public can assist a person with gambling problems. Some of the statements may seem contradictory or controversial; however, we have included them because they reflect the wide range of people's beliefs about the best ways to provide help to someone with gambling problems. It is important to note that we do not necessarily agree with these statements; we have included them because we do not believe that we should decide what the best practice is in this area. Rather, we have invited you to be a member of the expert panel to help develop a set of guidelines that reflect current expert opinion.

You will note that there is a place for you to add comments at the end of each section. This is so you can suggest any additional statements you think are important to giving help to a person with gambling problems. These statements will then go into the second questionnaire to be rated by all of the expert panels.

Instructions

Please complete the questionnaire by rating each statement **according to how important you believe it is for inclusion in the guidelines** for helping a person with gambling problems. Please keep in mind that the guidelines will be used by the general public. The statements need to be rated according to their importance for someone **without a counselling or clinical background** helping a person with gambling problems.

This questionnaire should take approximately 60 minutes to complete. You can complete the survey in two or more sittings. Your answers are saved when you click *'Next'* at the bottom of a page. This marks your page and you can begin again at a later date on the next page. **Please be aware that once you have logged on and started responding you must complete the questionnaire on the same computer.**

Consent to participate in this research

Consent to participate

It is important for you to know that participation in this study is completely voluntary. You are not under any obligation to participate and you can withdraw at any time.

We would like to thank you for your time and effort and encourage you to provide us with feedback on this process.

Best Wishes,

The Mental Health First Aid Research Team

To participate in this research you must:

• **Have a lived experience of gambling problems**, but are currently recovered and have experience in an advocacy or peer support role,

OR

• **Are a family member or friend** who has assisted a person with a gambling problem and have experience in an advocacy or peer support role,

OR

• Have a minimum of 2 years' experience **specialising in research on or treatment of** problem gambling.

*1. Please tick the appropriate answer.

◯ Yes, I meet this criteria.

◯ No, I do not meet this criteria.

Thank you!

Thank you for your time and willingness to participate, however you are not eligible to participate in this research. Please exit the survey now.

Consent to participate in this research (cont.)

*2. I understand that by submitting this survey I am giving my consent to participate in this study.

◯ Yes, I understand.

Overview of survey content

This survey is divided into the following section:

SECTION 1: Warning signs of a gambling problem

SECTION 2: Awareness of gambling and gambling problems

SECTION 3: Good communication skills

SECTION 4: First aid actions

Helping a person with gambling problems

Definitions of terms used in this survey

First aider refers to a concerned family member, friend, work colleague, or work supervisor who provides help to a person with gambling problems.

The person refers to the person with gambling problems or suspected gambling problems.

Gambling is the staking of money on uncertain events driven by chance.

Gambling problems are difficulties over time in limiting money or time spent on gambling, which leads to adverse consequences for the person, others, or for the community. This could include someone whose gambling problems are at a clinically diagnosable level.

Venue refers to a virtual or land-based location offering gambling or gaming activities with the chance to win money.

Gambling first aid is the help given to the person who is developing a gambling problem or experiencing a mental health crisis related to gambling. The assistance is given until appropriate professional help is received or until the crisis resolves.

Information about you

*3. Which best describes your area of expertise:

- ◯ I have a lived experience of gambling problems
- ◯ I am a family member or friend
- ◯ I have experience specialising in research on or treatment of problem gambling

*4. How old are you?

[ ]

*5. What is your gender?

- ◯ Female
- ◯ Male
- ◯ Other

*6. Please name all the relevant organisations you are affiliated with and your role within these organisations, e.g. professional organisations, peer support programs, advocacy groups.

[ ]

*7. Where do you live?

City/Town [ ]

State/Province [ ]

Country [ ]

Section 1: Warning signs

Helping a person with gambling problems

This section contains statements about the warning signs of gambling problems.

Please rate how important (from 'essential' to 'should not be included') you think it is that each statement be included in the guidelines.

*8. Behavioural Signs

The first aider should be aware that the following behavioural signs indicate that a person may have gambling problems:

	Essential	Important	Don't know/Depends	Unimportant	Should not be included
The person frequently thinks and talks about gambling.	○	○	○	○	○
The person frequently uses gambling terms in everyday conversation, e.g. bet, favourite.	○	○	○	○	○
The person complains of boredom when they are not gambling.	○	○	○	○	○
The person is restless when they are not gambling.	○	○	○	○	○
The person's family finds evidence of regular gambling, e.g. gambling receipts, Internet browser history, bank statements.	○	○	○	○	○
The person lies to cover up gambling activities.	○	○	○	○	○
The person gambles every day of the week.	○	○	○	○	○
The person increases the number or range of venues they gamble in.	○	○	○	○	○
The person increases the time they spend gambling.	○	○	○	○	○
The person is evasive about gambling losses.	○	○	○	○	○
The person expresses thoughts about gambling that are not realistic, e.g. "It must be my turn for a large win."	○	○	○	○	○
The person boasts about gambling wins.	○	○	○	○	○
The person continues to gamble despite promising to stop.	○	○	○	○	○
The person has tried unsuccessfully to control, cut back or stop gambling.	○	○	○	○	○
The person has a pattern of gambling for longer than intended.	○	○	○	○	○
The person is away from home for unexplained periods of time.	○	○	○	○	○
The person lies to fund gambling.	○	○	○	○	○
The person commits illegal acts to fund	○	○	○	○	○

gambling, e.g. embezzlement, fraud.					
The person steals from family or friends to fund gambling.	○	○	○	○	○
The person has legal problems related to gambling.	○	○	○	○	○
Before gambling, the person expresses a fear that they may miss an opportunity to win, e.g. "If I don't gamble today my lucky numbers will come up and I will miss out."	○	○	○	○	○
Before gambling, the person is over-confident or expresses fantasies about winning.	○	○	○	○	○
Before gambling, the person expresses excitement in anticipation of gambling.	○	○	○	○	○
After winning, the person expresses relief.	○	○	○	○	○
After winning, the person expresses a conviction that the win was the result of their skill.	○	○	○	○	○
After winning, the person appears to have an elated mood.	○	○	○	○	○
The person celebrates their wins by gambling more.	○	○	○	○	○
After losing, the person expresses fear of others finding out.	○	○	○	○	○
After losing, the person expresses worry over where they will get money to cover living expenses.	○	○	○	○	○
After losing, the person expresses anger towards themselves.	○	○	○	○	○
After losing, the person uses alcohol to forget about gambling problems.	○	○	○	○	○
The person has a pattern of returning to gambling in order to recover losses.	○	○	○	○	○

9. Please provide any additional behavioural signs:

Section 1: Warning signs (cont.)

This section contains statements about the warning signs of gambling problems.

Please rate how important (from ‘essential’ to ‘should not be included’) you think it is that each statement be included in the guidelines.

***10. Financial Signs**

The first aider should be aware that the following financial signs indicate that a person may have gambling problems:

	Essential	Important	Don't know/Depends	Unimportant	Should not be included
The family believes they can't trust the person with money.	○	○	○	○	○
Valuables disappear (and may reappear) without explanation.	○	○	○	○	○
The person is bad-tempered about money.	○	○	○	○	○
The person hides financial statements.	○	○	○	○	○
The person is secretive about money.	○	○	○	○	○
The person cannot explain missing amounts of money from the house or bank accounts.	○	○	○	○	○
The person constantly swaps money from one account to another.	○	○	○	○	○
The person has numerous personal loans.	○	○	○	○	○
The family finds hidden and unexplained money, e.g. money in a separate bank account, cash hidden in a drawer.	○	○	○	○	○
The person's family has to hide money from the person in order to cover living expenses.	○	○	○	○	○
The person or their family has experienced financial hardship as a result of gambling.	○	○	○	○	○
The person is consistently late in paying bills or misses payments entirely.	○	○	○	○	○
The person complains about mounting debts.	○	○	○	○	○
The person has a history of defaulting on payments.	○	○	○	○	○
The person or their family is frequently bothered by debt collectors.	○	○	○	○	○
The person owes money to a loan shark.	○	○	○	○	○
The person takes on extra jobs or works for overtime pay, but has no money to show for it.	○	○	○	○	○
The person cashes in investments or other assets early.	○	○	○	○	○
The person is frequently short of money.	○	○	○	○	○
The person has a pattern of unexplained loss of money.	○	○	○	○	○
	○	○	○	○	○

The person has a history of reporting that their money has been stolen or lost.	◯	◯	◯	◯	◯
The person borrows money to gamble or to pay gambling debts.	◯	◯	◯	◯	◯
The person has a pattern of spending all available funds in an episode of gambling.	◯	◯	◯	◯	◯
Over time, the person increases the amount of money spent on gambling.	◯	◯	◯	◯	◯
The person believes that gambling will solve their financial difficulties or bring material wealth.	◯	◯	◯	◯	◯
The person does not want to spend money on anything but gambling.	◯	◯	◯	◯	◯

11. Please provide any additional financial signs:

Section 1: Warning signs

This section contains statements about the warning signs of gambling problems.

Please rate how important (from ‘essential’ to ‘should not be included’) you think it is that each statement be included in the guidelines.

*12. Mental and physical health signs

The first aider should be aware that the following mental and physical health signs indicate that a person may have gambling problems:

	Essential	Important	Don't know/Depends	Unimportant	Should not be included
The person has experienced negative emotions as a result of gambling, e.g. sadness, anxiety, stress, anger.	◯	◯	◯	◯	◯
The person's family has experienced negative emotions as a result of gambling, e.g. sadness, anxiety, stress, anger.	◯	◯	◯	◯	◯
The person's mental health has been negatively affected as a result of gambling.	◯	◯	◯	◯	◯
The mental health of the person's family has been negatively affected as a result of gambling.	◯	◯	◯	◯	◯
The person experiences remorse or feels depressed after gambling.	◯	◯	◯	◯	◯
The person's self-esteem is tied to their gambling wins and losses.	◯	◯	◯	◯	◯
The person gambles to escape problems.	◯	◯	◯	◯	◯
The person's physical health has been negatively affected as a result of gambling.	◯	◯	◯	◯	◯
The physical health of the person's family has been negatively affected as a result of gambling.	◯	◯	◯	◯	◯

13. Please provide any additional mental or physical health signs:

Section 1: Warning signs (cont.)

This section contains statements about the warning signs of gambling problems.

Please rate how important (from ‘essential’ to ‘should not be included’) you think it is that each statement be included in the guidelines.

*14. Social signs

The first aider should be aware that the following social signs signs indicate that a person may have gambling problems:

	Essential	Important	Don't know/Depends	Unimportant	Should not be included
The person's relationships have been negatively affected as a result of gambling.	○	○	○	○	○
The person's or their family's social life has been negatively affected as a result of gambling.	○	○	○	○	○
The person's partner is threatening to leave or break up the family due to the gambling.	○	○	○	○	○
The person has conflicts with others about money.	○	○	○	○	○
The person is criticised by others for their gambling.	○	○	○	○	○
The person's reputation has suffered due to gambling.	○	○	○	○	○

15. Please provide any additional social signs:

Section 1: Warning signs (cont.)

This section contains statements about the warning signs of gambling problems.

Please rate how important (from ‘essential’ to ‘should not be included’) you think it is that each statement be included in the guidelines.

Helping a person with gambling problems

***16. Signs evident while gambling**

The first aider should be aware that the following signs indicate that a person may have gambling problems:

	Essential	Important	Don't know/Depends	Unimportant	Should not be included
The person gambles for three or more hours without a break of at least 15 minutes.	○	○	○	○	○
The person gambles for five or more hours without a break of at least 15 minutes.	○	○	○	○	○
The person focuses so intensely on gambling that they don't react to what is going on around them.	○	○	○	○	○
While gambling, the person avoids contact with others or communicates very little with anyone else.	○	○	○	○	○
The person bets above the minimum per spin most of the time.	○	○	○	○	○
The person has a significant change in their gambling expenditure pattern while gambling, e.g. sudden increases in size of bets.	○	○	○	○	○
The person stays on to gamble after friends leave the venue.	○	○	○	○	○
The person stops gambling only when the venue is closing.	○	○	○	○	○
The person finds it difficult to stop gambling at closing time.	○	○	○	○	○
The person regularly starts gambling as soon as the venue is open.	○	○	○	○	○
The person asks venue staff to not let other people know that they are there.	○	○	○	○	○
The person has friends or relatives call or arrive at the venue asking if the person is still there.	○	○	○	○	○
The person withdraws cash two or more times while at a gambling venue.	○	○	○	○	○
The person borrows money from others while at the venue.	○	○	○	○	○
The person asks for a loan or credit from the venue.	○	○	○	○	○
The person gambles with large wins.	○	○	○	○	○
The person leaves the venue to find money so that they can continue gambling.	○	○	○	○	○
The person rummages around in purse or wallet for additional money to gamble.	○	○	○	○	○
	○	○	○	○	○

The person gambles until all the money they have with them is used up.					
The person is shaking while gambling.					
The person sweats a lot while gambling.					
The person looks nervous or edgy, e.g. leg switching, bites lip continuously.					
The person displays anger, e.g. swears to themselves, grunts, kicks or strikes gaming machine.					
The person looks very sad or depressed after gambling.					
The person cries after losing a lot of money.					
The person sits with their head in their hands after losing.					
The person blames venues or gaming machines for losing.					
The person swears at or complains to staff about losing.					
The person groans repeatedly while gambling.					
The person shows significant changes in mood during a gambling session.					
The person appears to avoid the cashier or appears evasive by only using cash machine.					
The person gambles after having drunk a lot of alcohol.					
The person plays the gaming machine very roughly and aggressively, e.g. with fists or slaps.					
The person stands over other players while waiting for his or her favourite gaming machine.					
The person becomes angry if someone takes the person's favourite gaming machine or spot in the venue.					
After winning on gaming machines, the person plays on quickly without stopping to listen to the music or jingle.					
The person gambles on 2 or more gaming machines at once (where this is allowed).					
The person plays faster than most (e.g. inserts large numbers of coins into the gaming machine very rapidly, presses the buttons very rapidly so that the spin rate is very fast).					
The person performs ritualistic or superstitious routines while gambling, e.g. compulsively rubs belly of machine or screen while playing.					

The person rushes from one gaming machine or gaming table to another. ◯ ◯ ◯ ◯ ◯

17. Please provide any additional signs that may be apparent while the person is gambling:

Section 1: Warning signs (cont.)

This section contains statements about the warning signs of gambling problems.

Please rate how important (from ‘essential’ to ‘should not be included’) you think it is that each statement be included in the guidelines.

Helping a person with gambling problems

*18. Signs evident while at work

The first aider should be aware that the following signs at work indicate that a person may have gambling problems:

	Essential	Important	Don't know/Depends	Unimportant	Should not be included
The person gambles during work time.	○	○	○	○	○
The person reads newspaper and sports literature related to gambling at work.	○	○	○	○	○
The person repeatedly violates company gambling policy.	○	○	○	○	○
The person's ability to work or study has been negatively affected as a result of gambling.	○	○	○	○	○
The person is away from work for unexplained periods of time.	○	○	○	○	○
The person's use of company money seems suspicious or inappropriate.	○	○	○	○	○
The person takes sick days as soon as they are available.	○	○	○	○	○
The person has a role in financial management and takes their annual leave in isolated days rather than in blocks of time, so that others do not take over their responsibilities while they are away.	○	○	○	○	○
The person offers to collect money and place bets for others.	○	○	○	○	○
The person plans holidays around gambling.	○	○	○	○	○
The person runs workplace gambling activities, sports pools, tipping competitions or sweeps.	○	○	○	○	○
The person organises workplace social events that revolve around gambling.	○	○	○	○	○
The person receives visits or phone calls from debt collectors while at work.	○	○	○	○	○
The person asks for advances on their pay.	○	○	○	○	○
The person borrows money from co-workers.	○	○	○	○	○
The person argues with co-workers over money owed.	○	○	○	○	○
The person steals items from their company to resell.	○	○	○	○	○
The person sells items at work (either personal or stolen items).	○	○	○	○	○
The person arranges to have personal financial statements sent to work rather than home.	○	○	○	○	○

19. Please provide any additional signs that may be apparent while the person is at work:

Section 2: Awareness about gambling and gambling problems

This section contains statements about what the first aider needs to know about gambling problems.

Please rate how important (from 'essential' to 'should not be included') you think it is that each statement be included in the guidelines.

First aider awareness about gambling problems

***20. The first aider should understand that it is important to help a person with gambling problems because of the possible significant consequences, e.g. relationship breakdown, criminal sanctions, loss of employment, suicide, poor physical and mental health.**

Essential	Important	Don't know/Depends	Unimportant	Should not be included
○	○	○	○	○

***21. The first aider should learn all they can about gambling problems by reading about them.**

Essential	Important	Don't know/Depends	Unimportant	Should not be included
○	○	○	○	○

***22. The first aider should learn all they can about gambling problems by attending a support group.**

Essential	Important	Don't know/Depends	Unimportant	Should not be included
○	○	○	○	○

***23. The first aider should be aware of the risk factors for problem gambling.**

Essential	Important	Don't know/Depends	Unimportant	Should not be included
○	○	○	○	○

***24. The first aider should know that some types of gambling can cause more problems than others (e.g. gaming machines), however any form of gambling can become a problem.**

Essential	Important	Don't know/Depends	Unimportant	Should not be included
○	○	○	○	○

***25. The first aider should know about the common motivations for gambling, e.g. to win money, or because it is fun, exciting or social.**

Essential	Important	Don't know/Depends	Unimportant	Should not be included
○	○	○	○	○

Helping a person with gambling problems

***26. The first aider should know about the common motivations for gambling in people with gambling problems, e.g. chasing losses, escaping negative emotions, building self-esteem.**

Essential	Important	Don't know/Depends	Unimportant	Should not be included
○	○	○	○	○

***27. The first aider should be aware that the person may see gambling as the only way to make up for losses.**

Essential	Important	Don't know/Depends	Unimportant	Should not be included
○	○	○	○	○

***28. The first aider should know that the person may feel the need to gamble with increasing amounts of money as a way to achieve desired feelings of excitement.**

Essential	Important	Don't know/Depends	Unimportant	Should not be included
○	○	○	○	○

***29. The first aider should know that gambling problems are mental health problems.**

Essential	Important	Don't know/Depends	Unimportant	Should not be included
○	○	○	○	○

***30. The first aider should be aware that people with gambling problems are likely to have other mental health problems, e.g. depression, anxiety, alcohol or other drug use problems.**

Essential	Important	Don't know/Depends	Unimportant	Should not be included
○	○	○	○	○

31. Are there any additional statements you think are important to giving help to a person with gambling problems? Please write your suggestions in the box provided.

Section 2: Awareness about gambling and gambling problems (cont.)

This section contains statements about what the first aider needs to know about the person with gambling problems.

Please rate how important (from 'essential' to 'should not be included') you think it is that each statement be included in the guidelines.

The person's awareness about their gambling problems

***32. The first aider should be aware that a person with gambling problems may not see them as a problem, even though it may be obvious to those around them.**

Essential	Important	Don't know/Depends	Unimportant	Should not be included
○	○	○	○	○

Helping a person with gambling problems

***33. The first aider should be aware that a person with gambling problems may not see them as a problem, until they experience a crisis that they cannot solve themselves.**

Essential | Important | Don't know/Depends | Unimportant | Should not be included

***34. The first aider should be aware that the person may go through cycles of awareness and denial that their gambling is a problem.**

Essential | Important | Don't know/Depends | Unimportant | Should not be included

***35. The first aider should know that the person's family member or partner may not be aware or able to admit that the person has gambling problems.**

Essential | Important | Don't know/Depends | Unimportant | Should not be included

36. Are there any additional statements you think are important to giving help to a person with gambling problems? Please write your suggestions in the box provided.

Section 2: Awareness about gambling and gambling problems (cont.)

This section contains statements about what the first aider needs to know about treatment and recovery for gambling problems.

Please rate how important (from 'essential' to 'should not be included') you think it is that each statement be included in the guidelines.

Awareness about treatment and recovery

***37. The first aider should know that a gambling problem is an illness that cannot be cured, but that the person can learn to abstain.**

Essential | Important | Don't know/Depends | Unimportant | Should not be included

***38. The first aider should know that the goals of treatment could be either abstinence from gambling or restricting gambling activities.**

Essential | Important | Don't know/Depends | Unimportant | Should not be included

***39. The first aider should know that gambling problems can be successfully treated.**

Essential | Important | Don't know/Depends | Unimportant | Should not be included

Helping a person with gambling problems

***40. The first aider should not assume that a gambling problem is a phase the person is likely to pass through.**

Essential	Important	Don't know/Depends	Unimportant	Should not be included
○	○	○	○	○

***41. The first aider should be aware that recovery from gambling problems is a slow process.**

Essential	Important	Don't know/Depends	Unimportant	Should not be included
○	○	○	○	○

***42. The first aider should know that support from family and friends can assist the person's recovery.**

Essential	Important	Don't know/Depends	Unimportant	Should not be included
○	○	○	○	○

***43. The first aider should encourage the person to seek treatment with their partner or a family member.**

Essential	Important	Don't know/Depends	Unimportant	Should not be included
○	○	○	○	○

***44. The first aider should know the Stages of Change Model and its implications for helping the person.**

Essential	Important	Don't know/Depends	Unimportant	Should not be included
○	○	○	○	○

***45. The first aider should be aware that the person may have tried and failed repeatedly to control, cut back or stop gambling.**

Essential	Important	Don't know/Depends	Unimportant	Should not be included
○	○	○	○	○

***46. The first aider should be familiar with the effective treatments available for gambling problems.**

Essential	Important	Don't know/Depends	Unimportant	Should not be included
○	○	○	○	○

47. Are there any additional statements you think are important to giving help to a person with gambling problems? Please write your suggestions in the box provided.

Section 2: Awareness about gambling and gambling problems (cont.)

This section contains statements about what the first aider needs to know about treatment and recovery for gambling problems.

Please rate how important (from 'essential' to 'should not be included') you think it is that each statement be included in the guidelines.

Awareness about gambling resources

***48. The first aider should be aware that the person may need to access various services, including medical help, gambling counselling, legal services, mental health services, financial counselling, vocational rehabilitation or social assistance.**

Essential	Important	Don't know/Depends	Unimportant	Should not be included
◯	◯	◯	◯	◯

***49. The first aider should be aware of resources to help the person with their gambling problems.**

Essential	Important	Don't know/Depends	Unimportant	Should not be included
◯	◯	◯	◯	◯

***50. The first aider should inform the person about available sources of help.**

Essential	Important	Don't know/Depends	Unimportant	Should not be included
◯	◯	◯	◯	◯

***51. The first aider should encourage the person to seek the type of help that is most appropriate for them.**

Essential	Important	Don't know/Depends	Unimportant	Should not be included
◯	◯	◯	◯	◯

***52. The first aider should be aware of resources that can help the person to manage their financial difficulties.**

Essential	Important	Don't know/Depends	Unimportant	Should not be included
◯	◯	◯	◯	◯

***53. The first aider should be aware of any local mechanisms for excluding people with gambling problems from venues.**

Essential	Important	Don't know/Depends	Unimportant	Should not be included
◯	◯	◯	◯	◯

54. Are there any additional statements you think are important to giving help to a person with gambling problems? Please write your suggestions in the box provided.

Section 3: Good communication skills

This section contains statements about how to communicate with the person about gambling problems.

Please rate how important (from 'essential' to 'should not be included') you think it is that each statement be included in the guidelines.

Preparing to talk with the person

Helping a person with gambling problems

***55. The first aider should be prepared for the full range of responses they may encounter when talking with the person about their gambling problems, e.g. relief, anger.**

Essential	Important	Don't know/Depends	Unimportant	Should not be included
○	○	○	○	○

***56. The first aider should prepare for approaching the person about their gambling problems by speaking in advance to a professional who is knowledgeable about gambling problems.**

Essential	Important	Don't know/Depends	Unimportant	Should not be included
○	○	○	○	○

***57. The first aider should practice what they want to say beforehand, e.g. writing it out or practicing with another person.**

Essential	Important	Don't know/Depends	Unimportant	Should not be included
○	○	○	○	○

***58. The first aider should consider writing down their concerns and presenting these to the person.**

Essential	Important	Don't know/Depends	Unimportant	Should not be included
○	○	○	○	○

***59. The first aider should prepare by having information about available help for gambling problems.**

Essential	Important	Don't know/Depends	Unimportant	Should not be included
○	○	○	○	○

***60. The first aider should choose an appropriate place to talk, e.g. private, away from distractions and interruptions.**

Essential	Important	Don't know/Depends	Unimportant	Should not be included
○	○	○	○	○

***61. The first aider should pick a time when both the first aider and the person are feeling well, rather than tired or upset.**

Essential	Important	Don't know/Depends	Unimportant	Should not be included
○	○	○	○	○

***62. The first aider should choose a time when there is enough time to talk.**

Essential	Important	Don't know/Depends	Unimportant	Should not be included
○	○	○	○	○

63. Are there any additional statements you think are important to giving help to a person with gambling problems? Please write your suggestions in the box provided.

Section 3: Good communication skills (cont.)

This section contains statements about how to communicate with the person about gambling problems.

Please rate how important (from 'essential' to 'should not be included') you think it is that each statement be included in the guidelines.

Talking with the person

*64. When approaching the person to discuss their gambling problems, the first aider should:

	Essential	Important	Don't know/Depends	Unimportant	Should not be included
Try to talk about the gambling problems in a calm and rational manner.	○	○	○	○	○
State some positive things about the person and their relationship with the first aider.	○	○	○	○	○
Use 'I' statements rather than 'you' statements, e.g. "I feel worried when I don't know when you are coming home or how much money you will have spent," rather than "You upset me when you are late and have spent all our money."	○	○	○	○	○
Talk about what they have noticed, e.g. that the person spends a lot of time at gambling venues.	○	○	○	○	○
Focus on specific concerns about the person's behaviour and its impact.	○	○	○	○	○
Focus on the impact of the person's gambling behaviour rather than on the person themselves as the problem.	○	○	○	○	○
Focus on what the person is going to do about their gambling problems.	○	○	○	○	○
Ask the person for their perspective and allow them to tell their story.	○	○	○	○	○
Validate the person's experience and feelings, e.g. "I understand that gambling is important to you."	○	○	○	○	○

65. Are there any additional statements you think are important to giving help to a person with gambling problems? Please write your suggestions in the box provided.

Section 3: Good communication skills (cont.)

This section contains statements about how to communicate with the person about gambling problems.

Please rate how important (from 'essential' to 'should not be included') you think it is that each statement be included in the guidelines.

Talking with the person (cont.)

*66. The first aider should avoid:

	Essential	Important	Don't know/Depends	Unimportant	Should not be included
Lecturing the person about their gambling problems.	○	○	○	○	○
Interrogating the person about their gambling activities.	○	○	○	○	○
Trying to control the person by threatening, bribing, crying, or nagging.	○	○	○	○	○
Arguing with the person.	○	○	○	○	○
Verbally or physically attacking the person.	○	○	○	○	○

67. Are there any additional statements you think are important to giving help to a person with gambling problems? Please write your suggestions in the box provided.

Section 3: Good communication skills (cont.)

This section contains statements about how to communicate with the person about gambling problems.

Please rate how important (from 'essential' to 'should not be included') you think it is that each statement be included in the guidelines.

Talking with the person (cont.)

*68. The first aider should be aware the person may make promises that they are unable to keep.

Essential	Important	Don't know/Depends	Unimportant	Should not be included
○	○	○	○	○

*69. The first aider should be aware that the person may use denial, minimisation, rationalisation or blaming when approached by the first aider about their gambling problems.

Essential	Important	Don't know/Depends	Unimportant	Should not be included
○	○	○	○	○

*70. The first aider should be aware that the person may lie about their gambling problems.

Essential	Important	Don't know/Depends	Unimportant	Should not be included
○	○	○	○	○

Helping a person with gambling problems

***71. The first aider should keep private any discussions with the person.**

Essential	Important	Don't know/Depends	Unimportant	Should not be included
○	○	○	○	○

***72. The first aider should keep private any discussions with the person, unless there is concern about the safety of the person or others.**

Essential	Important	Don't know/Depends	Unimportant	Should not be included
○	○	○	○	○

***73. The first aider should be aware that a person with gambling problems may feel ashamed or embarrassed.**

Essential	Important	Don't know/Depends	Unimportant	Should not be included
○	○	○	○	○

***74. The first aider should be aware the person may not want to talk to them about their gambling problems because of feelings of shame.**

Essential	Important	Don't know/Depends	Unimportant	Should not be included
○	○	○	○	○

***75. If the person does not want to talk to the first aider about their gambling problems, the first aider should tell them about services that are available to help them.**

Essential	Important	Don't know/Depends	Unimportant	Should not be included
○	○	○	○	○

76. Are there any additional statements you think are important to giving help to a person with gambling problems? Please write your suggestions in the box provided.

Section 3: Good communication skills (cont.)

This section contains statements about how to communicate with the person about gambling problems.

Please rate how important (from ‘essential’ to ‘should not be included’) you think it is that each statement be included in the guidelines.

Dealing with difficulties when interacting with the person

***77. If the person doesn't want to talk about the gambling problems right now, the first aider should let the person know that they are available to talk when the person is ready.**

Essential	Important	Don't know/Depends	Unimportant	Should not be included
○	○	○	○	○

***78. If the person tries to change the subject, the first aider should keep the conversation focused on the gambling problems.**

Essential	Important	Don't know/Depends	Unimportant	Should not be included
○	○	○	○	○

***79. If the person denies or rationalises their gambling problems, the first aider should be prepared to offer evidence of the problem, e.g. credit card or bank statements.**

Essential	Important	Don't know/Depends	Unimportant	Should not be included
○	○	○	○	○

***80. If the person continues to deny or rationalise the problems, the first aider should end the discussion and try again at another time.**

Essential	Important	Don't know/Depends	Unimportant	Should not be included
○	○	○	○	○

***81. If the conversation ends without a resolution, the first aider should try to get agreement from the person on another time to continue the discussion.**

Essential	Important	Don't know/Depends	Unimportant	Should not be included
○	○	○	○	○

***82. If the conversation becomes unproductive or aggressive, the first aider should end the discussion and try again at another time.**

Essential	Important	Don't know/Depends	Unimportant	Should not be included
○	○	○	○	○

***83. If the first aider finds it difficult to approach the person about their gambling problems, they should consider anonymously sending them information about gambling problems.**

Essential	Important	Don't know/Depends	Unimportant	Should not be included
○	○	○	○	○

84. Are there any additional statements you think are important to giving help to a person with gambling problems? Please write your suggestions in the box provided.

Section 4: Gambling first aid actions

This section contains statements about what the first aider should do to support someone with gambling problems.

Please rate how important (from ‘essential’ to ‘should not be included’) you think it is that each statement be included in the guidelines.

Encouraging help-seeking

Helping a person with gambling problems

***85. The first aider should talk with the person as soon as they suspect the person has gambling problems, as early intervention may reduce the negative impact.**

Essential	Important	Don't know/Depends	Unimportant	Should not be included
○	○	○	○	○

***86. The first aider should encourage the person to seek professional help for their gambling problems.**

Essential	Important	Don't know/Depends	Unimportant	Should not be included
○	○	○	○	○

***87. If the person asks, the first aider should go with the person to an appointment for professional help.**

Essential	Important	Don't know/Depends	Unimportant	Should not be included
○	○	○	○	○

***88. The first aider should encourage the person to attend a support group.**

Essential	Important	Don't know/Depends	Unimportant	Should not be included
○	○	○	○	○

***89. The first aider should encourage the person to use self-help strategies.**

Essential	Important	Don't know/Depends	Unimportant	Should not be included
○	○	○	○	○

***90. The first aider should <u>not</u> attempt to force the person to seek professional help or attend a support group.**

Essential	Important	Don't know/Depends	Unimportant	Should not be included
○	○	○	○	○

91. Are there any additional statements you think are important to giving help to a person with gambling problems? Please write your suggestions in the box provided.

Section 4: Gambling first aid actions (cont.)

This section contains statements about what the first aider should do to support someone with gambling problems.

Please rate how important (from 'essential' to 'should not be included') you think it is that each statement be included in the guidelines.

<u>Encouraging help-seeking (cont.)</u>

Helping a person with gambling problems

***92. The first aider can encourage help-seeking by pointing out that:**

	Essential	Important	Don't know/Depends	Unimportant	Should not be included
Many people with gambling problems have benefited from professional help.	○	○	○	○	○
Many people with gambling problems have benefited from support groups.	○	○	○	○	○
Many people with gambling problems have benefited from self-help strategies.	○	○	○	○	○
Seeking help is not a sign of weakness.	○	○	○	○	○
Seeking help is a sensible thing to do when experiencing a problem, like seeing a doctor for a health problem.	○	○	○	○	○
The sooner the problem is addressed the easier it is to overcome.	○	○	○	○	○
Any professional help will be confidential.	○	○	○	○	○

93. Are there any additional statements you think are important to giving help to a person with gambling problems? Please write your suggestions in the box provided.

Section 4: Gambling first aid actions (cont.)

This section contains statements about what the first aider should do to support someone with gambling problems.

Please rate how important (from 'essential' to 'should not be included') you think it is that each statement be included in the guidelines.

Encouraging help-seeking (cont.)

***94. The first aider should help the person take responsibility for their gambling problems by allowing the person to experience the consequences of their gambling, e.g. dealing with debt, experiencing problems with relationships.**

Essential	Important	Don't know/Depends	Unimportant	Should not be included
○	○	○	○	○

***95. If the first aider pays the person's debts or expenses, they should insist that the person gets professional help.**

Essential	Important	Don't know/Depends	Unimportant	Should not be included
○	○	○	○	○

***96. If the first aider pays the person's debts or expenses, they should make arrangements for the person to pay back the loan, even if it is a small amount each week.**

Essential	Important	Don't know/Depends	Unimportant	Should not be included
○	○	○	○	○

Helping a person with gambling problems

***97. The first aider should work with the person to agree on acceptable behaviours, e.g. talking to a professional, staying within agreed spending limits.**

Essential	Important	Don't know/Depends	Unimportant	Should not be included
◯	◯	◯	◯	◯

***98. The first aider should put any agreement about behaviour change in writing and ask the person to sign the document.**

Essential	Important	Don't know/Depends	Unimportant	Should not be included
◯	◯	◯	◯	◯

99. Are there any additional statements you think are important to giving help to a person with gambling problems? Please write your suggestions in the box provided.

Section 4: Gambling first aid actions (cont.)

This section contains statements about what the first aider should do to support someone with gambling problems.

Please rate how important (from 'essential' to 'should not be included') you think it is that each statement be included in the guidelines.

Supporting change

***100. The first aider should know about the common false beliefs that can lead to gambling problems, e.g. belief that they can beat the system or superstitions about luck.**

Essential	Important	Don't know/Depends	Unimportant	Should not be included
◯	◯	◯	◯	◯

***101. The first aider should tell the person about the common false beliefs that can lead to gambling problems, e.g. belief that they can beat the system or superstitions about luck.**

Essential	Important	Don't know/Depends	Unimportant	Should not be included
◯	◯	◯	◯	◯

***102. The first aider should discourage people, such as family and friends from taking responsibility for the consequences of the gambling problems.**

Essential	Important	Don't know/Depends	Unimportant	Should not be included
◯	◯	◯	◯	◯

103. Are there any additional statements you think are important to giving help to a person with gambling problems? Please write your suggestions in the box provided.

Section 4: Gambling first aid actions (cont.)

This section contains statements about what the first aider should do to support someone with gambling problems.

Please rate how important (from 'essential' to 'should not be included') you think it is that each statement be included in the guidelines.

Supporting change (cont.)

*104. The first aider should not:

	Essential	Important	Don't know/Depends	Unimportant	Should not be included
Give the person money.	○	○	○	○	○
Give the person money, except to avoid severe consequences, e.g. legal sanctions.	○	○	○	○	○
Pay the person's debt.	○	○	○	○	○
Pay the person's debt, except to avoid severe consequences, e.g. legal sanctions.	○	○	○	○	○
Cover the person's basic living expenses.	○	○	○	○	○
Cover the person's basic living expenses, except to avoid severe consequences, e.g. homelessness.	○	○	○	○	○
Deny the person's basic needs, e.g. food or shelter.	○	○	○	○	○
Take responsibility away from the person for managing their financial problems.	○	○	○	○	○
Cover for the person's behaviours.	○	○	○	○	○
Lie to cover for the person's behaviours.	○	○	○	○	○
Deny to themselves or others that the person has a problem.	○	○	○	○	○
Expect the person to be rational about their gambling.	○	○	○	○	○
Expect the person to immediately control their gambling.	○	○	○	○	○
Accept blame for the person's gambling problems.	○	○	○	○	○
Justify the person's gambling, to themselves or others.	○	○	○	○	○
Minimise the person's gambling problems in order to avoid conflict.	○	○	○	○	○
Go gambling with the person	○	○	○	○	○
Drop off or pick up the person from gambling activities.	○	○	○	○	○
Tell the person to just stop gambling.	○	○	○	○	○
Use shame or guilt to force the person into change.	○	○	○	○	○

105. Are there any additional statements you think are important to giving help to a person with gambling problems? Please write your suggestions in the box provided.

Section 4: Gambling first aid actions (cont.)

This section contains statements about what the first aider should do to support someone with gambling problems.

Please rate how important (from 'essential' to 'should not be included') you think it is that each statement be included in the guidelines.

If the person does not want to change their gambling behaviours

***106. If the person does not want to change their gambling behaviours, the first aider should ask the person if gambling and its consequences are getting in the way of them living the life they want to live.**

Essential	Important	Don't know/Depends	Unimportant	Should not be included
○	○	○	○	○

***107. If the person is unwilling to seek professional help, the first aider should set limits around what behaviours they are willing and unwilling to accept from the person.**

Essential	Important	Don't know/Depends	Unimportant	Should not be included
○	○	○	○	○

***108. The first aider should not sever the relationship or distance themselves from the person.**

Essential	Important	Don't know/Depends	Unimportant	Should not be included
○	○	○	○	○

***109. If the person does not follow through with the recommendations to get treatment and asks for financial or practical help in the future, the first aider should refuse unless the person agrees to treatment.**

Essential	Important	Don't know/Depends	Unimportant	Should not be included
○	○	○	○	○

***110. The first aider should not use ultimatums.**

Essential	Important	Don't know/Depends	Unimportant	Should not be included
○	○	○	○	○

***111. The first aider should not threaten consequences for the person's gambling behaviour that they are not prepared to carry out.**

Essential	Important	Don't know/Depends	Unimportant	Should not be included
○	○	○	○	○

Helping a person with gambling problems

112. Are there any additional statements you think are important to giving help to a person with gambling problems? Please write your suggestions in the box provided.

Section 4: Gambling first aid actions (cont.)

This section contains statements about what the first aider should do to support someone with gambling problems.

Please rate how important (from ‘essential’ to ‘should not be included’) you think it is that each statement be included in the guidelines.

Reducing the negative impact of gambling

***113. If the person decides to continue gambling, the first aider should encourage them to reduce the negative impact of gambling by:**

	Essential	Important	Don't know/Depends	Unimportant	Should not be included
Limiting time spent on gambling.	○	○	○	○	○
Limiting money spent on gambling.	○	○	○	○	○
Only gambling with money that the person can afford to lose.	○	○	○	○	○
Restricting their gambling to activities where the person has greater control over their behaviour.	○	○	○	○	○
Balancing time spent on gambling with other activities.	○	○	○	○	○
Keeping a record of gambling wins and losses.	○	○	○	○	○
Eating before gambling.	○	○	○	○	○
Stopping gambling after a win.	○	○	○	○	○
Taking regular breaks while gambling.	○	○	○	○	○
Gambling with someone who limits their gambling.	○	○	○	○	○

114. Are there any additional statements you think are important to giving help to a person with gambling problems? Please write your suggestions in the box provided.

Section 4: Gambling first aid actions (cont.)

This section contains statements about what the first aider should do to support someone with gambling problems.

Please rate how important (from ‘essential’ to ‘should not be included’) you think it is that each statement be included in the guidelines.

Reducing the negative impact of gambling (cont.)

***115. If the person decides to continue gambling, the first aider should encourage them to reduce the negative impact of gambling by not:**

	Essential	Important	Don't know/Depends	Unimportant	Should not be included
Gambling to earn money or pay debts.	○	○	○	○	○
Trying to win back gambling losses.	○	○	○	○	○
Using borrowed money to gamble.	○	○	○	○	○
Using personal investments or savings to gamble.	○	○	○	○	○
Taking cash or bank cards to access extra money while gambling.	○	○	○	○	○
Gambling when their judgment is impaired by alcohol or other drugs.	○	○	○	○	○
Gambling to escape from problems or feelings.	○	○	○	○	○
Gambling when angry or upset.	○	○	○	○	○
Gambling alone.	○	○	○	○	○

116. Are there any additional statements you think are important to giving help to a person with gambling problems? Please write your suggestions in the box provided.

Section 4: Gambling first aid actions (cont.)

This section contains statements about what the first aider should do to support someone with gambling problems.

Please rate how important (from 'essential' to 'should not be included') you think it is that each statement be included in the guidelines.

Helping the person who wants to change their gambling behaviours

***117. If the person decides to seek professional help, the first aider should offer to support the person during this, e.g. talk with the person about issues, skills and exercises that come out of therapy.**

Essential	Important	Don't know/Depends	Unimportant	Should not be included
○	○	○	○	○

***118. If the person decides to use self-help strategies, the first aider should offer to support the person during this.**

Essential	Important	Don't know/Depends	Unimportant	Should not be included
○	○	○	○	○

Helping a person with gambling problems

***119. The first aider should help the person list the advantages and disadvantages of gambling.**

Essential	Important	Don't know/Depends	Unimportant	Should not be included
○	○	○	○	○

***120. The first aider should encourage the person to write down, on a daily basis, the negative consequences of gambling.**

Essential	Important	Don't know/Depends	Unimportant	Should not be included
○	○	○	○	○

***121. The first aider should encourage the person to write down, on a daily basis, the positive consequences of <u>not</u> gambling.**

Essential	Important	Don't know/Depends	Unimportant	Should not be included
○	○	○	○	○

***122. The first aider should help the person make a list of strategies that can help them change their gambling behaviours.**

Essential	Important	Don't know/Depends	Unimportant	Should not be included
○	○	○	○	○

***123. If the person is attempting to change their gambling behaviours, the first aider should focus on the future, rather than past mistakes.**

Essential	Important	Don't know/Depends	Unimportant	Should not be included
○	○	○	○	○

***124. If the person is attempting to change their gambling behaviours, the first aider should note any positive behavioural changes and congratulate the person on these.**

Essential	Important	Don't know/Depends	Unimportant	Should not be included
○	○	○	○	○

***125. If the person sets a budget and asks for help sticking to it, the first aider should support them in this.**

Essential	Important	Don't know/Depends	Unimportant	Should not be included
○	○	○	○	○

***126. The first aider should be aware that the person who has stopped or reduced their gambling may experience a gap in their life that gambling used to fill, e.g. reduction in social activities.**

Essential	Important	Don't know/Depends	Unimportant	Should not be included
○	○	○	○	○

***127. The first aider should suggest activities that they can do with the person that do not involve gambling, e.g. going to the movies or to a restaurant, reconnecting with family and friends.**

Essential	Important	Don't know/Depends	Unimportant	Should not be included
○	○	○	○	○

Helping a person with gambling problems

***128. If the person is experiencing anxiety, anger, stress, depression or boredom, the first aider should provide social support, as these may be triggers for worsening of gambling problems.**

Essential	Important	Don't know/Depends	Unimportant	Should not be included
○	○	○	○	○

***129. The first aider should discuss with the person possible strategies for handling gambling urges and encourage the person to use them.**

Essential	Important	Don't know/Depends	Unimportant	Should not be included
○	○	○	○	○

***130. If the person gambles online, the first aider should encourage them to use software programs that block certain sites, restrict access time or that monitor and report all activity from a computer.**

Essential	Important	Don't know/Depends	Unimportant	Should not be included
○	○	○	○	○

Section 4: Gambling first aid actions (cont.)

This section contains statements about what the first aider should do to support someone with gambling problems.

Please rate how important (from ‘essential’ to ‘should not be included’) you think it is that each statement be included in the guidelines.

Helping the person who wants to change their gambling behaviours (cont.)

***131. The first aider should encourage the person to do the following:**

	Essential	Important	Don't know/Depends	Unimportant	Should not be included
Accept that they will not win back past gambling losses.	○	○	○	○	○
Refrain from all forms of gambling.	○	○	○	○	○
Reduce the amount of time and money spent on gambling.	○	○	○	○	○
Tell those close to them about every aspect of their gambling problem including all of their debts.	○	○	○	○	○
Avoid spending time with people who are associated with gambling activities.	○	○	○	○	○
Regularly read a self-help book or website about changing their gambling behaviour.	○	○	○	○	○
Seek support from family, friends or others to help them change their gambling behaviour.	○	○	○	○	○

Helping a person with gambling problems

***132. The first aider should be aware that although relapse is a problem, a relapse does not indicate that the person cannot recover.**

Essential	Important	Don't know/Depends	Unimportant	Should not be included
○	○	○	○	○

***133. If the person has a relapse, the first aider should tell them that this is not a sign of long-term failure of recovery.**

Essential	Important	Don't know/Depends	Unimportant	Should not be included
○	○	○	○	○

134. Are there any additional statements you think are important to giving help to a person with gambling problems? Please write your suggestions in the box provided.

Section 4: Gambling first aid actions (cont.)

This section contains statements about what the first aider should do to support someone with gambling problems.

Please rate how important (from 'essential' to 'should not be included') you think it is that each statement be included in the guidelines.

Financial strategies

***135. The first aider should work with the person to address financial problems.**

Essential	Important	Don't know/Depends	Unimportant	Should not be included
○	○	○	○	○

***136. In order to limit access to money for gambling, the first aider should ask the person to consider the following:**

	Essential	Important	Don't know/Depends	Unimportant	Should not be included
Allowing someone else to manage their finances, e.g. partner or other family member.	○	○	○	○	○
Setting up accounts and loans so that they require a second signature.	○	○	○	○	○
Receiving an allowance from a family member so that the majority of the money can be used for household expenses.	○	○	○	○	○
Paying all critical household expenses before paying off gambling debts.	○	○	○	○	○

137. Are there any additional statements you think are important to giving help to a person with gambling problems? Please write your suggestions in the box provided.

Section 4: Gambling first aid actions (cont.)

This section contains statements about what the first aider should do to support someone with gambling problems.

Please rate how important (from 'essential' to 'should not be included') you think it is that each statement be included in the guidelines.

Interventions

An intervention is when a group of people who care about the person meet with the person to point out the behaviours that are causing problems and ask them to stop. An intervention includes a discussion around the consequences for the person if they do not get help for their gambling problem.

***138. The first aider should not organise an intervention.**

Essential	Important	Don't know/Depends	Unimportant	Should not be included
○	○	○	○	○

***139. The first aider should consider organising an intervention to help encourage the person to get professional help for their gambling problems.**

Essential	Important	Don't know/Depends	Unimportant	Should not be included
○	○	○	○	○

140. Are there any additional statements you think are important to giving help to a person with gambling problems? Please write your suggestions in the box provided.

Helping a person with gambling problems

***141. If the first aider decides to arrange an intervention, they should:**

	Essential	Important	Don't know/Depends	Unimportant	Should not be included
Consider engaging the help of a professional when organising an intervention.	○	○	○	○	○
Include friends and family who they know will be able to support the person in their recovery.	○	○	○	○	○
Select a location for the intervention that is not too familiar or comfortable for the person.	○	○	○	○	○
Organise the intervention in such a way that it is a surprise for the person.	○	○	○	○	○
Plan the goals and content of the intervention.	○	○	○	○	○
Organise the intervention in a way that the person feels supported and cared for rather than punished or shamed.	○	○	○	○	○
Practice the intervention with someone who is trained in conducting interventions (if available).	○	○	○	○	○
Describe each unacceptable behaviour.	○	○	○	○	○
Be specific about the time and place of each behaviour.	○	○	○	○	○
Describe the feelings each incident aroused in the first aider.	○	○	○	○	○
Only list the behaviours that the first aider has observed, not what they have been told.	○	○	○	○	○

142. Are there any additional statements you think are important to giving help to a person with gambling problems? Please write your suggestions in the box provided.

Section 4: Gambling first aid actions (cont.)

This section contains statements about what the first aider should do to support someone with gambling problems.

Please rate how important (from 'essential' to 'should not be included') you think it is that each statement be included in the guidelines.

When there are concerns for safety

***143. The first aider should be aware that suicidal thoughts and behaviours are more common in people with gambling problems.**

Essential	Important	Don't know/Depends	Unimportant	Should not be included
○	○	○	○	○

***144. The first aider should be aware that the person may see suicide as a way to avoid difficult confrontations with loved ones or creditors.**

Essential	Important	Don't know/Depends	Unimportant	Should not be included
○	○	○	○	○

***145. The first aider should be aware that the person may see suicide as a viable solution to financial problems due to life insurance payouts.**

Essential	Important	Don't know/Depends	Unimportant	Should not be included
○	○	○	○	○

***146. The first aider should be aware of the Mental Health First Aid Guidelines for how to help someone with suicidal thoughts or behaviours.**

Essential	Important	Don't know/Depends	Unimportant	Should not be included
○	○	○	○	○

***147. If the first aider is afraid that the person may harm themselves or someone else, the first aider should seek professional support before taking action to deal with the person's gambling problems.**

Essential	Important	Don't know/Depends	Unimportant	Should not be included
○	○	○	○	○

***148. The first aider should act to protect any children who are being neglected as a result of the person's gambling.**

Essential	Important	Don't know/Depends	Unimportant	Should not be included
○	○	○	○	○

***149. If the person is involved with loan sharks, the first aider should consider consulting the police.**

Essential	Important	Don't know/Depends	Unimportant	Should not be included
○	○	○	○	○

***150. If the person is involved with loan sharks, the first aider should encourage the person to consult the police.**

Essential	Important	Don't know/Depends	Unimportant	Should not be included
○	○	○	○	○

***151. If the person is involved in illegal activities related to their gambling, the first aider should:**

	Essential	Important	Don't know/Depends	Unimportant	Should not be included
Encourage the person to stop these activities.	○	○	○	○	○
Encourage the person to seek legal help.	○	○	○	○	○
Encourage the person to confess to the police.	○	○	○	○	○
Consult the police.	○	○	○	○	○

Helping a person with gambling problems

***152. The first aider should be aware that supporting a person with a gambling problem can be difficult and should know how to access support for themselves.**

Essential	Important	Don't know/Depends	Unimportant	Should not be included
○	○	○	○	○

153. Are there any additional statements you think are important to giving help to a person with gambling problems? Please write your suggestions in the box provided.

Thank you!

Thank you for your time!

That is the end of the first round survey. Thank you very much for your contribution.

If participating in this survey has caused you distress and you wish to talk to someone about this, you can contact any of the following:

Australia: Lifeline on 13 11 14

New Zealand: Lifeline Aotearoa on 0800 543 354

UK: Samaritans on 08457 909090

Republic of Ireland: Samaritans on 116 123

USA: National Suicide prevention Lifeline on 1800 273 TALK (8255)

Canada: National Suicide prevention Lifeline on 1800 273 TALK (8255)

By pressing the *'Next'* button your responses will be registered with our survey software. Once all panel members have lodged their responses, we will collate the data and send you a report on the findings and the second survey.

Kind Regards,

The Mental Health First Aid Research Team

Helping a person with gambling problems- Round 2

Information about this survey

How this questionnaire was developed

The statements in this questionnaire were derived from the results of the Round 1 survey. You will note that each statement is marked as either a new or rerate item. New items were derived from the comments provided in the first survey. An item is rerated when 80% or more of the panel members from 1 of the groups rated it as essential or important **AND** if 70%–79% of panel members from the other panel rated it as essential or important.

Instructions

Please complete the questionnaire by rating each statement **according to how important you believe it is for inclusion in the guidelines** for helping a person with gambling problems. Please keep in mind that the guidelines will be used by the general public. The statements need to be rated according to their importance for someone **without a counselling or clinical background** helping a person with gambling problems.

This questionnaire should take approximately 60 minutes to complete. You can complete the survey in two or more sittings. Your answers are saved when you click *'Next'* at the bottom of a page. This marks your page and you can begin again at a later date on the next page. **Please be aware that once you have logged on and started responding you must complete the questionnaire on the same computer.**

***1. Please provide your name so I can verify who is eligible to complete Round 3 and therefore is eligible for the payment.**

Helping a person with gambling problems- Round 2

Definitions of terms used in this survey

First aider refers to a concerned family member, friend, work colleague, or work supervisor who provides help to a person with gambling problems.

The person refers to the person with gambling problems or suspected gambling problems.

Gambling is the staking of money on uncertain events driven by chance.

Gambling problems are difficulties over time in limiting money or time spent on gambling, which leads to adverse consequences for the person, others, or for the community. This could include someone whose gambling problems are at a clinically diagnosable level.

Venue refers to a virtual or land-based location offering gambling or gaming activities with the chance to win money.

Gambling first aid is the help given to the person who is developing a gambling problem or experiencing a mental health crisis related to gambling. The assistance is given until appropriate professional help is received or until the crisis resolves.

Helping a person with gambling problems- Round 2

Section 1: Warning signs

This section contains statements about the warning signs of gambling problems.

Please rate how important (from 'essential' to 'should not be included') you think it is that each statement be included in the guidelines.

*** 2. The first aider should give the person a list of the signs of gambling problems and ask the person to consider if any of the signs apply to them. (New)**

Essential	Important	Don't know/Depends	Unimportant	Should not be included
○	○	○	○	○

*** 3. Behavioural Signs**

The first aider should be aware that the following behavioural signs indicate that a person may have gambling problems:

	Essential	Important	Don't know/Depends	Unimportant	Should not be included
1. The person expresses guilt about their gambling (New).	○	○	○	○	○
2. The person is evasive or defensive when questioned about missed events or responsibilities. (New)	○	○	○	○	○
3. The person becomes defensive or angry when asked about their gambling. (New)	○	○	○	○	○
4. The person blames others for their gambling or its consequences. (New)	○	○	○	○	○
5. The person neglects the basic care of their children. (New)	○	○	○	○	○
6. The person breaks promises to their children about buying them things or spending time with them due to gambling activities. (New)	○	○	○	○	○
7. The person argues with their partner when the partner spends money on household necessities. (New)	○	○	○	○	○
8. The person causes arguments so they can leave home and gamble. (New)	○	○	○	○	○
9. The person makes excuses not to attend family events. (New)	○	○	○	○	○
10. The person checks sports scores or racing results frequently, e.g. online, mobile device. (New)	○	○	○	○	○
11. The person gambles rather than doing things they previously enjoyed. (New)	○	○	○	○	○
12. The person reports that they cannot recall periods when they were gambling. (New)	○	○	○	○	○
13. The person expresses a strong desire	○	○	○	○	○

or craving to gamble. (New)	◯	◯	◯	◯	◯
14. The person reports dreaming about gambling. (New)	◯	◯	◯	◯	◯
15. The person gambles every day of the week. (Rerate)	◯	◯	◯	◯	◯
16. The person gambles almost every day. (New)	◯	◯	◯	◯	◯
17. The person increases the number or range of venues they gamble in. (Rerate)	◯	◯	◯	◯	◯
18. The person boasts about gambling wins. (Rerate)	◯	◯	◯	◯	◯
19. Before gambling, the person expresses a fear that they may miss an opportunity to win, e.g. "If I don't gamble today my lucky numbers will come up and I will miss out." (Rerate)	◯	◯	◯	◯	◯
20. Before gambling, the person is over-confident or expresses fantasies about winning. (Rerate)	◯	◯	◯	◯	◯
21. After a gambling loss, the person expresses anger towards family or friends. (New)	◯	◯	◯	◯	◯
22. After a gambling loss, the person expresses hopelessness. (New)	◯	◯	◯	◯	◯
23. After a gambling loss, the person engages in risky behaviour. (New)	◯	◯	◯	◯	◯

Helping a person with gambling problems- Round 2

Section 1: Warning signs (cont.)

This section contains statements about the warning signs of gambling problems.

Please rate how important (from ‘essential’ to ‘should not be included’) you think it is that each statement be included in the guidelines.

*4. Financial Signs

The first aider should be aware that the following financial signs indicate that a person may have gambling problems:

	Essential	Important	Don't know/Depends	Unimportant	Should not be included
1. The family believes they can't trust the person with money. (Rerate)	○	○	○	○	○
2. The person has numerous personal loans. (Rerate)	○	○	○	○	○
3. The person has a history of defaulting on payments. (Rerate)	○	○	○	○	○
4. The person or their family is frequently bothered by debt collectors. (Rerate)	○	○	○	○	○
5. The person cashes in investments or other assets early. (Rerate)	○	○	○	○	○
6. The person makes promises to pay back family and friends but never does so. (New)	○	○	○	○	○
7. The person is aware of all the ATM/money machines close to the venue. (New)	○	○	○	○	○
8. The person increases their usage of or acquires additional credit cards. (New)	○	○	○	○	○
9. The person alternates between having no money and have abundant amounts of money. (New)	○	○	○	○	○

Helping a person with gambling problems- Round 2

Section 1: Warning signs

This section contains statements about the warning signs of gambling problems.

Please rate how important (from ‘essential’ to ‘should not be included’) you think it is that each statement be included in the guidelines.

*5. Mental and physical health signs

The first aider should be aware that the following mental and physical health signs indicate that a person may have gambling problems:

	Essential	Important	Don't know/Depends	Unimportant	Should not be included
1. The person does not look after their health as a result of their gambling activities, e.g. does not take medication or eat a healthy diet. (New)	◯	◯	◯	◯	◯
2. The person does not look after personal hygiene due to gambling problems. (New)	◯	◯	◯	◯	◯
3. The person has frequent unexplained mood swings. (New)	◯	◯	◯	◯	◯

Helping a person with gambling problems- Round 2

Section 1: Warning signs (cont.)

This section contains statements about the warning signs of gambling problems.

Please rate how important (from ‘essential’ to ‘should not be included’) you think it is that each statement be included in the guidelines.

*6. Social signs

The first aider should be aware that the following social signs signs indicate that a person may have gambling problems:

	Essential	Important	Don't know/Depends	Unimportant	Should not be included
1. The person's reputation has suffered due to gambling. (Rerate)	◯	◯	◯	◯	◯
2. The person disappears from social events where gambling is also available, in order to gamble. (New)	◯	◯	◯	◯	◯
3. The person is unable to be emotionally present or involved in social situations because they are pre-occupied with gambling. (New)	◯	◯	◯	◯	◯
4. The person isolates themselves because of gambling. (New)	◯	◯	◯	◯	◯

Helping a person with gambling problems- Round 2

Section 1: Warning signs (cont.)

This section contains statements about the warning signs of gambling problems.

Please rate how important (from 'essential' to 'should not be included') you think it is that each statement be included in the guidelines.

*7. Signs evident while gambling

The first aider should be aware that the following signs indicate that a person may have gambling problems:

	Essential	Important	Don't know/Depends	Unimportant	Should not be included
1. The person gambles for three or more hours without a break of at least 15 minutes. (Rerate)	○	○	○	○	○
2. While gambling, the person avoids contact with others or communicates very little with anyone else. (Rerate)	○	○	○	○	○
3. The person avoids taking phone calls while at a gambling venue. (new)	○	○	○	○	○
4. The person has a significant change in their gambling expenditure pattern while gambling, e.g. sudden increases in size of bets. (Rerate)	○	○	○	○	○
5. The person asks venue staff to not let other people know that they are there. (Rerate)	○	○	○	○	○
6. The person borrows money from others while at the venue. (Rerate)	○	○	○	○	○
7. The person asks for a loan or credit from the venue. (Rerate)	○	○	○	○	○
8. The person gambles until all the money they have with them is used up. (Rerate)	○	○	○	○	○
9. The person cries after losing a lot of money. (Rerate)	○	○	○	○	○
10. The person blames venues or gaming machines for losing. (Rerate)	○	○	○	○	○
11. The person becomes angry if someone takes the person's favourite gaming machine or spot in the venue. (Rerate)	○	○	○	○	○
12. The person plays faster than most (e.g. inserts large numbers of coins into the gaming machine very rapidly, presses the buttons very rapidly so that the spin rate is very fast). (Rerate)	○	○	○	○	○
13. The person is particular about reserving their machine while they take a break. (New)	○	○	○	○	○
14. The person offers to show others how	○	○	○	○	○

to win at gambling. (New)	○	○	○	○	○
15. The person often gambles on their own. (New)	○	○	○	○	○

Helping a person with gambling problems- Round 2

Section 1: Warning signs (cont.)

This section contains statements about the warning signs of gambling problems.

Please rate how important (from ‘essential’ to ‘should not be included’) you think it is that each statement be included in the guidelines.

*8. Signs evident while at work

The first aider should be aware that the following signs at work indicate that a person may have gambling problems:

	Essential	Important	Don't know/Depends	Unimportant	Should not be included
1. The person repeatedly violates company gambling policy. (Rerate)	○	○	○	○	○
2. The person's use of company money seems suspicious or inappropriate. (Rerate)	○	○	○	○	○
3. The person receives visits or phone calls from debt collectors while at work. (Rerate)	○	○	○	○	○
4. The person borrows money from co-workers. (Rerate)	○	○	○	○	○
5. The person argues with co-workers over money owed. (Rerate)	○	○	○	○	○
6. The person sells items at work (either personal or stolen items). (Rerate)	○	○	○	○	○

Helping a person with gambling problems- Round 2

Section 2: Awareness about gambling and gambling problems

This section contains statements about what the first aider needs to know about gambling problems.

Please rate how important (from 'essential' to 'should not be included') you think it is that each statement be included in the guidelines.

First aider awareness about gambling problems

***9. The first aider should learn all they can about gambling problems by reading about them. (Rerate)**

Essential	Important	Don't know/Depends	Unimportant	Should not be included
○	○	○	○	○

***10. The first aider should know about the common motivations for gambling, e.g. to win money, or because it is fun, exciting or social. (Rerate)**

Essential	Important	Don't know/Depends	Unimportant	Should not be included
○	○	○	○	○

***11. The first aider should know that gambling problems are mental health problems. (Rerate)**

Essential	Important	Don't know/Depends	Unimportant	Should not be included
○	○	○	○	○

***12. The first aider should be aware that there is an increased risk of violence in families affected by gambling problems. (New)**

Essential	Important	Don't know/Depends	Unimportant	Should not be included
○	○	○	○	○

Section 2: Awareness about gambling and gambling problems (cont.)

This section contains statements about what the first aider needs to know about treatment and recovery for gambling problems.

Please rate how important (from ‘essential’ to ‘should not be included’) you think it is that each statement be included in the guidelines.

Awareness about treatment and recovery

***13. The first aider should be aware that recovery from gambling problems is a slow process. (Rerate)**

Essential	Important	Don't know/Depends	Unimportant	Should not be included
○	○	○	○	○

***14. The first aider should know the Stages of Change Model and its implications for helping the person. (Rerate)**

Essential	Important	Don't know/Depends	Unimportant	Should not be included
○	○	○	○	○

***15. The first aider should be aware that it is not necessary for the person's recovery that they tell all their family and friends about their gambling problems. (New)**

Essential	Important	Don't know/Depends	Unimportant	Should not be included
○	○	○	○	○

***16. The first aider should be aware that they are not personally responsible for 'fixing' the person's gambling problems. (New)**

Essential	Important	Don't know/Depends	Unimportant	Should not be included
○	○	○	○	○

Section 2: Awareness about gambling and gambling problems (cont.)

This section contains statements about what the first aider needs to know about treatment and recovery for gambling problems.

Please rate how important (from ‘essential’ to ‘should not be included’) you think it is that each statement be included in the guidelines.

Awareness about gambling resources

***17. The first aider should be aware of any local mechanisms for excluding people with gambling problems from venues. (Rerate)**

Essential	Important	Don't know/Depends	Unimportant	Should not be included
◯	◯	◯	◯	◯

***18. The first aider should provide information about self-help resources and support groups. (New)**

Essential	Important	Don't know/Depends	Unimportant	Should not be included
◯	◯	◯	◯	◯

***19. The first aider should work together with the person to identify the most appropriate supports for the person. (New)**

Essential	Important	Don't know/Depends	Unimportant	Should not be included
◯	◯	◯	◯	◯

***20. If the person is from a culturally or linguistically diverse background, the first aider should suggest culturally appropriate services, where available. (New)**

Essential	Important	Don't know/Depends	Unimportant	Should not be included
◯	◯	◯	◯	◯

***21. If the first aider gives the person details about gambling help services, they should call the person to follow-up and check if they contacted the service. (New)**

Essential	Important	Don't know/Depends	Unimportant	Should not be included
◯	◯	◯	◯	◯

Helping a person with gambling problems- Round 2

Section 3: Good communication skills

This section contains statements about how to communicate with the person about gambling problems.

Please rate how important (from ‘essential’ to ‘should not be included’) you think it is that each statement be included in the guidelines.

Preparing to talk with the person

***22. The first aider should pick a time when both the first aider and the person are feeling well, rather than tired or upset. (Rerate)**

Essential ○ Important ○ Don't know/Depends ○ Unimportant ○ Should not be included ○

***23. The first aider should know that it may be difficult to find an ideal time to talk with the person, therefore they should just talk with the person rather than wait for the 'perfect' timing. (New)**

Essential ○ Important ○ Don't know/Depends ○ Unimportant ○ Should not be included ○

***24. The first aider should look for opportunities to talk to the person about their gambling in everyday interactions. (New)**

Essential ○ Important ○ Don't know/Depends ○ Unimportant ○ Should not be included ○

***25. The first aider should know that sometimes the best time to talk to the person is when they are in crisis following a gambling occasion. (New)**

Essential ○ Important ○ Don't know/Depends ○ Unimportant ○ Should not be included ○

***26. The first aider should not approach the person alone if there is a possibility that the person may become violent. (New)**

Essential ○ Important ○ Don't know/Depends ○ Unimportant ○ Should not be included ○

***27. If the person does not show up for an arranged meeting, the first aider should not take this personally, but rather schedule another time. (New)**

Essential ○ Important ○ Don't know/Depends ○ Unimportant ○ Should not be included ○

***28. If the first aider knows someone who has recovered from a gambling problem, they should enlist this person's help when they make the initial approach about their concerns. (New)**

Essential ○ Important ○ Don't know/Depends ○ Unimportant ○ Should not be included ○

Section 3: Good communication skills (cont.)

This section contains statements about how to communicate with the person about gambling problems.

Please rate how important (from 'essential' to 'should not be included') you think it is that each statement be included in the guidelines.

Talking with the person (cont.)

***29. The first aider should keep private any discussions with the person. (Rerate)**

Essential	Important	Don't know/Depends	Unimportant	Should not be included
○	○	○	○	○

***30. The first aider should share relevant personal experiences with the person. (New)**

Essential	Important	Don't know/Depends	Unimportant	Should not be included
○	○	○	○	○

***31. The first aider should avoid trying to convince the person to quit gambling. (New)**

Essential	Important	Don't know/Depends	Unimportant	Should not be included
○	○	○	○	○

***32. Rather than telling the person what to do, the first aider should make suggestions, e.g. "Would you be comfortable seeing a gambling counsellor" rather than "You should go see a gambling counsellor". (New)**

Essential	Important	Don't know/Depends	Unimportant	Should not be included
○	○	○	○	○

***33. The first aider should encourage the person to talk to others who have experienced gambling problems, as they may relate better. (New)**

Essential	Important	Don't know/Depends	Unimportant	Should not be included
○	○	○	○	○

***34. The first aider should explain to the person that they will try not to be judgmental and ask the person to tell them if they are. (New)**

Essential	Important	Don't know/Depends	Unimportant	Should not be included
○	○	○	○	○

***35. When talking to the person about their gambling problems, the first aider should use empathy and compassion. (New)**

Essential	Important	Don't know/Depends	Unimportant	Should not be included
○	○	○	○	○

***36. The first aider should listen to the person without passing judgement. (New)**

Essential	Important	Don't know/Depends	Unimportant	Should not be included
○	○	○	○	○

***37. If the first aider has not experienced gambling problems themselves, they should tell the person that although they do not understand what the person is going through, they are available to help them if the person would like. (New)**

Essential	Important	Don't know/Depends	Unimportant	Should not be included
○	○	○	○	○

***38. The first aider should give the person enough time to tell their story because this will help the person to open up and trust the first aider. (New)**

Essential	Important	Don't know/Depends	Unimportant	Should not be included
○	○	○	○	○

***39. If the first aider thinks that their ability to help the person is impeded by any negative attitudes towards the person's gambling or gambling in general, they should suggest the person talks with someone else. (New)**

Essential	Important	Don't know/Depends	Unimportant	Should not be included
○	○	○	○	○

Section 3: Good communication skills (cont.)

This section contains statements about how to communicate with the person about gambling problems.

Please rate how important (from 'essential' to 'should not be included') you think it is that each statement be included in the guidelines.

Dealing with difficulties when interacting with the person

***40. If the person denies they have a problem, the first aider should consider gathering evidence to back up their concerns for a future conversation. (New)**

Essential	Important	Don't know/Depends	Unimportant	Should not be included
○	○	○	○	○

***41. The first aider should avoid letting the person engage in long explanations or excuses for their gambling behaviour, but instead focus on potential solutions. (New)**

Essential	Important	Don't know/Depends	Unimportant	Should not be included
○	○	○	○	○

***42. If the person denies they have a problem, the first aider should consider enlisting the help of another person to back up what they are saying. (New)**

Essential	Important	Don't know/Depends	Unimportant	Should not be included
○	○	○	○	○

***43. If the person does not want to talk to the first aider about their gambling problems, the first aider should consider enlisting someone who has a good relationship with the person to talk with them. (New)**

Essential	Important	Don't know/Depends	Unimportant	Should not be included
○	○	○	○	○

Helping a person with gambling problems- Round 2

Section 4: Gambling first aid actions

This section contains statements about what the first aider should do to support someone with gambling problems.

Please rate how important (from 'essential' to 'should not be included') you think it is that each statement be included in the guidelines.

Encouraging help-seeking

***44. The first aider should talk with the person as soon as they suspect the person has gambling problems, as early intervention may reduce the negative impact. (Rerate)**

Essential	Important	Don't know/Depends	Unimportant	Should not be included
○	○	○	○	○

***45. The first aider should tell the person that it is possible to recover from gambling problems. (New)**

Essential	Important	Don't know/Depends	Unimportant	Should not be included
○	○	○	○	○

***46. The first aider should <u>not</u> attempt to force the person to seek professional help or attend a support group. (Rerate)**

Essential	Important	Don't know/Depends	Unimportant	Should not be included
○	○	○	○	○

***47. The first aider should work with the person to agree on acceptable behaviours, e.g. talking to a professional, staying within agreed spending limits. (Rerate)**

Essential	Important	Don't know/Depends	Unimportant	Should not be included
○	○	○	○	○

***48. If the person is reluctant to seek help, the first aider should tell the person that most people react in a supportive way when gambling problems are disclosed. (New)**

Essential	Important	Don't know/Depends	Unimportant	Should not be included
○	○	○	○	○

***49. If the person asks, the first aider should go with the person to an appointment for professional help, but should not be present during the consultation. (New)**

Essential	Important	Don't know/Depends	Unimportant	Should not be included
○	○	○	○	○

***50. If the person decides to seek professional help, the first aider should offer to support the person during this, e.g. talk with the person about issues, skills and exercises that come out of therapy. (Rerate)**

Essential	Important	Don't know/Depends	Unimportant	Should not be included
○	○	○	○	○

Helping a person with gambling problems- Round 2

Section 4: Gambling first aid actions (cont.)

This section contains statements about what the first aider should do to support someone with gambling problems.

Please rate how important (from 'essential' to 'should not be included') you think it is that each statement be included in the guidelines.

Supporting change

*51. The first aider should not:

	Essential	Important	Don't know/Depends	Unimportant	Should not be included
1. Give the person money. (Rerate)	◯	◯	◯	◯	◯
2. Go gambling with the person. (Rerate)	◯	◯	◯	◯	◯
3. Drop off or pick up the person from gambling activities. (Rerate)	◯	◯	◯	◯	◯
4. Tell the person to just stop gambling. (Rerate)	◯	◯	◯	◯	◯
5. Use shame or guilt to force the person into change. (Rerate)	◯	◯	◯	◯	◯

*52. The first aider should encourage the person to learn about the strategies that gambling providers use to keep people gambling and maximise profits, e.g. gaming machines are designed to keep people playing and spending money. (New)

Essential	Important	Don't know/Depends	Unimportant	Should not be included
◯	◯	◯	◯	◯

*53. The first aider should encourage the person to avoid going to gambling venues even if they are not going to gamble (e.g. going to a pub for a meal where gambling is available). (New)

Essential	Important	Don't know/Depends	Unimportant	Should not be included
◯	◯	◯	◯	◯

*54. The first aider should be clear about what they are willing to help the person with and what behaviours they will tolerate, although these boundaries can be revisited over time. (New)

Essential	Important	Don't know/Depends	Unimportant	Should not be included
◯	◯	◯	◯	◯

*55. The first aider should ask the person if there are any problems that have led them to increasing their gambling behaviours. (New)

Essential	Important	Don't know/Depends	Unimportant	Should not be included
◯	◯	◯	◯	◯

*56. The first aider should encourage the person to apologise to anyone who has been harmed by their gambling activities. (New)

Essential	Important	Don't know/Depends	Unimportant	Should not be included
◯	◯	◯	◯	◯

Section 4: Gambling first aid actions (cont.)

This section contains statements about what the first aider should do to support someone with gambling problems.

Please rate how important (from 'essential' to 'should not be included') you think it is that each statement be included in the guidelines.

If the person does not want to change their gambling behaviours

***57. The first aider should be aware that the person may not want to change their gambling behaviours. (New)**

Essential	Important	Don't know/Depends	Unimportant	Should not be included
◯	◯	◯	◯	◯

***58. The first aider should be aware that the person can only be helped if they are ready to change their gambling behaviours. (New)**

Essential	Important	Don't know/Depends	Unimportant	Should not be included
◯	◯	◯	◯	◯

***59. The first aider should tell the person that they can only be helped if they are ready to change their gambling behaviours. (New)**

Essential	Important	Don't know/Depends	Unimportant	Should not be included
◯	◯	◯	◯	◯

***60. If the person does not want to change their gambling behaviours, the first aider should ask the person if gambling and its consequences are getting in the way of them living the life they want to live. (Rerate)**

Essential	Important	Don't know/Depends	Unimportant	Should not be included
◯	◯	◯	◯	◯

***61. If the person does not want to change their gambling behaviours, the first aider should sensitively ask the person if gambling and its consequences are getting in the way of them living the life they want to live. (New)**

Essential	Important	Don't know/Depends	Unimportant	Should not be included
◯	◯	◯	◯	◯

***62. The first aider should tell the person that they are unlikely to win back their losses by gambling. (New)**

Essential	Important	Don't know/Depends	Unimportant	Should not be included
◯	◯	◯	◯	◯

***63. The first aider should tell the person that they will be available to help the person when they are ready to change their gambling behaviours. (New)**

Essential	Important	Don't know/Depends	Unimportant	Should not be included
◯	◯	◯	◯	◯

***64. The first aider should not threaten consequences for the person's gambling behaviour that they are not prepared to carry out. (Rerate)**

Essential	Important	Don't know/Depends	Unimportant	Should not be included
◯	◯	◯	◯	◯

Section 4: Gambling first aid actions (cont.)

This section contains statements about what the first aider should do to support someone with gambling problems.

Please rate how important (from 'essential' to 'should not be included') you think it is that each statement be included in the guidelines.

Reducing the negative impact of gambling

* 65. If the person decides to continue gambling, the first aider should encourage them to reduce the negative impact of gambling by:

	Essential	Important	Don't know/Depends	Unimportant	Should not be included
1. Only gambling with money that the person can afford to lose. (Rerate)	○	○	○	○	○
2. Restricting their gambling to activities where the person has greater control over their behaviour. (Rerate)	○	○	○	○	○
3. Balancing time spent on gambling with other activities. (Rerate)	○	○	○	○	○
4. Taking regular breaks while gambling. (Rerate)	○	○	○	○	○
5. Leaving bank cards or credit cards at home. (New)	○	○	○	○	○
6. Keeping a photo or other reminder of something they value, but that they are at risk of losing due to gambling (e.g. family, house), in their wallet. (New)	○	○	○	○	○
7. Gambling only when all debts are paid off. (New)	○	○	○	○	○

* 66. If the person decides to continue gambling, the first aider should discourage them from gambling with friends or family who have gambling problems. (New)

Essential	Important	Don't know/Depends	Unimportant	Should not be included
○	○	○	○	○

* 67. If the person decides to continue gambling, the first aider should discourage them from gambling alone. (Rerate)

Essential	Important	Don't know/Depends	Unimportant	Should not be included
○	○	○	○	○

Helping a person with gambling problems- Round 2

Section 4: Gambling first aid actions (cont.)

This section contains statements about what the first aider should do to support someone with gambling problems.

Please rate how important (from 'essential' to 'should not be included') you think it is that each statement be included in the guidelines.

Helping the person who wants to change their gambling behaviours

***68. The first aider should help the person make a list of strategies that can help them change their gambling behaviours. (Rerate)**

Essential	Important	Don't know/Depends	Unimportant	Should not be included
○	○	○	○	○

***69. If the person is attempting to change their gambling behaviours, the first aider should focus on the future, rather than past mistakes. (Rerate)**

Essential	Important	Don't know/Depends	Unimportant	Should not be included
○	○	○	○	○

***70. The first aider should encourage the person to avoid spending time with people who are associated with gambling activities. (Rerate)**

Essential	Important	Don't know/Depends	Unimportant	Should not be included
○	○	○	○	○

***71. The first aider should explain to the person that gambling problems took time to develop, so it may take them some time, and more than one attempt, to change their gambling behaviours. (New)**

Essential	Important	Don't know/Depends	Unimportant	Should not be included
○	○	○	○	○

***72. The first aider should discuss with the person possible strategies for handling gambling urges and encourage the person to use them. (Rerate)**

Essential	Important	Don't know/Depends	Unimportant	Should not be included
○	○	○	○	○

***73. <u>If asked by the person</u>, the first aider should discuss with them possible strategies for handling gambling urges and encourage the person to use them. (New)**

Essential	Important	Don't know/Depends	Unimportant	Should not be included
○	○	○	○	○

***74. The first aider should continue to offer support, even if the person has a relapse. (New)**

Essential	Important	Don't know/Depends	Unimportant	Should not be included
○	○	○	○	○

Section 4: Gambling first aid actions (cont.)

This section contains statements about what the first aider should do to support someone with gambling problems.

Please rate how important (from 'essential' to 'should not be included') you think it is that each statement be included in the guidelines.

Financial strategies

*75. In order to limit access to money for gambling, the first aider should ask the person to consider the following:

	Essential	Important	Don't know/Depends	Unimportant	Should not be included
1. Receiving an allowance from a family member so that the majority of the money can be used for household expenses. (Rerate)	◯	◯	◯	◯	◯
2. Having a trusted relative or close friend take temporary control of the person's access to funds. (New)	◯	◯	◯	◯	◯
3. Arranging that they have access to a limited amount of money each day that covers daily expenses, e.g. lunch, parking, coffee. (New)	◯	◯	◯	◯	◯
4. Paying all critical household expenses before paying off gambling debts. (Rerate)	◯	◯	◯	◯	◯

*76. The first aider should encourage the person to be transparent about finances with their partner or family, e.g. mutual access to bank and credit card records. (New)

Essential	Important	Don't know/Depends	Unimportant	Should not be included
◯	◯	◯	◯	◯

*77. If the first aider gives the person cash for a specific purpose, they should ask the person to provide a store receipt and return any change. (New)

Essential	Important	Don't know/Depends	Unimportant	Should not be included
◯	◯	◯	◯	◯

*78. The first aider should be aware that the person may experience a sense of relief if someone else takes control of their finances. (New)

Essential	Important	Don't know/Depends	Unimportant	Should not be included
◯	◯	◯	◯	◯

*79. If the person asks for money to help cover bills or debts, the first aider should refer them to a relief agency or financial counselling service, rather than giving them money. (New)

Essential	Important	Don't know/Depends	Unimportant	Should not be included
◯	◯	◯	◯	◯

***80. The first aider should know that if they choose to lend the person money, it is unlikely to be repaid. (New)**

Essential	Important	Don't know/Depends	Unimportant	Should not be included
○	○	○	○	○

Section 4: Gambling first aid actions (cont.)

This section contains statements about what the first aider should do to support someone with gambling problems.

Please rate how important (from 'essential' to 'should not be included') you think it is that each statement be included in the guidelines.

Interventions

An intervention is when a group of people who care about the person meet with the person to point out the behaviours that are causing problems and ask them to stop. An intervention includes a discussion around the consequences for the person if they do not get help for their gambling problem.

***81. The first aider should arrange a family meeting with the person to discuss the best way to resolve the gambling problems and to encourage help-seeking. (New)**

Essential	Important	Don't know/Depends	Unimportant	Should not be included
○	○	○	○	○

***82. If the first aider decides to arrange an intervention, they should consider engaging the help of a professional when organising an intervention. (Rerate)**

Essential	Important	Don't know/Depends	Unimportant	Should not be included
○	○	○	○	○

***83. If the first aider decides to arrange an intervention, they should Include friends and family who they know will be able to support the person in their recovery. (Rerate)**

Essential	Important	Don't know/Depends	Unimportant	Should not be included
○	○	○	○	○

***84. The first aider should only consider organising an intervention as a last resort. (New)**

Essential	Important	Don't know/Depends	Unimportant	Should not be included
○	○	○	○	○

Section 4: Gambling first aid actions (cont.)

This section contains statements about what the first aider should do to support someone with gambling problems.

Please rate how important (from 'essential' to 'should not be included') you think it is that each statement be included in the guidelines.

When there are concerns for safety

***85. If the person is involved in illegal activities related to their gambling, the first aider should encourage the person to seek legal help. (Rerate)**

Essential	Important	Don't know/Depends	Unimportant	Should not be included
◯	◯	◯	◯	◯

***86. The first aider should act to protect family members who may be at risk due to the person's gambling activities. (New)**

Essential	Important	Don't know/Depends	Unimportant	Should not be included
◯	◯	◯	◯	◯

***87. The first aider should encourage the person to contact anyone who has been harmed by any illegal activity due to the person's gambling activities, admit what they have done and face any consequences. (New)**

Essential	Important	Don't know/Depends	Unimportant	Should not be included
◯	◯	◯	◯	◯

Helping a person with gambling problems- Round 2

Thank you!

Thank you for your time!

That is the end of the Round 2 survey. Thank you very much for your contribution.

If participating in this survey has caused you distress and you wish to talk to someone about this, you can contact any of the following:

Australia: Lifeline on 13 11 14

New Zealand: Lifeline Aotearoa on 0800 543 354

UK: Samaritans on 08457 909090

Republic of Ireland: Samaritans on 116 123

USA: National Suicide prevention Lifeline on 1800 273 TALK (8255)

Canada: National Suicide prevention Lifeline on 1800 273 TALK (8255)

By pressing the *'Next'* button your responses will be registered with our survey software. Once all panel members have lodged their responses, we will collate the data and send you a report on the findings and the final survey.

Kind Regards,

The Mental Health First Aid Research Team

Information about this survey

How this questionnaire was developed

The statements in this questionnaire were derived from the results of the Round 2 survey. All of the items in this survey were 'new' items in the Round 2 survey that need to be rerated. An item is rerated when 80% or more of the panel members from 1 of the groups rated it as essential or important **AND** if 70%–79% of panel members from the other panel rated it as essential or important. **OR** if 70%-79% of both panels rated it as essential or important.

Instructions

Please complete the questionnaire by rating each statement **according to how important you believe it is for inclusion in the guidelines** for helping a person with gambling problems. Please keep in mind that the guidelines will be used by the general public. The statements need to be rated according to their importance for someone **without a counselling or clinical background** helping a person with gambling problems.

This questionnaire should take approximately 20 minutes to complete. You can complete the survey in two or more sittings. Your answers are saved when you click *'Next'* at the bottom of a page. This marks your page and you can begin again at a later date on the next page. **Please be aware that once you have logged on and started responding you must complete the questionnaire on the same computer.**

***1. Please provide your name so I can verify who is eligible for the payment.**

Definitions of terms used in this survey

First aider refers to a concerned family member, friend, work colleague, or work supervisor who provides help to a person with gambling problems.

The person refers to the person with gambling problems or suspected gambling problems.

Gambling is the staking of money on uncertain events driven by chance.

Gambling problems are difficulties over time in limiting money or time spent on gambling, which leads to adverse consequences for the person, others, or for the community. This could include someone whose gambling problems are at a clinically diagnosable level.

Venue refers to a virtual or land-based location offering gambling or gaming activities with the chance to win money.

Gambling first aid is the help given to the person who is developing a gambling problem or experiencing a mental health crisis related to gambling. The assistance is given until appropriate professional help is received or until the crisis resolves.

Section 1: Warning signs

This section contains statements about the warning signs of gambling problems.

Please rate how important (from ‘essential’ to ‘should not be included’) you think it is that each statement be included in the guidelines.

***2. The first aider should give the person a list of the signs of gambling problems and ask the person to consider if any of the signs apply to them.**

Essential	Important	Don't know/Depends	Unimportant	Should not be included
○	○	○	○	○

***3. Behavioural Signs**

The first aider should be aware that the following behavioural signs indicate that a person may have gambling problems:

	Essential	Important	Don't know/Depends	Unimportant	Should not be included
1. The person is evasive or defensive when questioned about missed events or responsibilities.	○	○	○	○	○
2. The person blames others for their gambling or its consequences.	○	○	○	○	○
3. The person checks sports scores or racing results frequently, e.g. online, mobile device.	○	○	○	○	○
4. The person gambles almost every day.	○	○	○	○	○
5. After a gambling loss, the person expresses anger towards family or friends.	○	○	○	○	○
6. After a gambling loss, the person engages in risky behaviour.	○	○	○	○	○

***4. Financial Signs**

The first aider should be aware that the following financial signs indicate that a person may have gambling problems:

	Essential	Important	Don't know/Depends	Unimportant	Should not be included
1. The person alternates between having no money and having abundant amounts of money.	○	○	○	○	○

*5. Mental and physical health signs

The first aider should be aware that the following mental and physical health signs indicate that a person may have gambling problems:

	Essential	Important	Don't know/Depends	Unimportant	Should not be included
1. The person does not look after personal hygiene due to gambling problems.	◯	◯	◯	◯	◯

*6. Signs evident while gambling

The first aider should be aware that the following signs indicate that a person may have gambling problems:

	Essential	Important	Don't know/Depends	Unimportant	Should not be included
1. The person often gambles on their own.	◯	◯	◯	◯	◯

Section 3: Good communication skills

This section contains statements about how to communicate with the person about gambling problems.

Please rate how important (from ‘essential’ to ‘should not be included’) you think it is that each statement be included in the guidelines.

Talking with the person

*7. The first aider should explain to the person that they will try not to be judgmental and ask the person to tell them if they are.

Essential	Important	Don't know/Depends	Unimportant	Should not be included
◯	◯	◯	◯	◯

Section 4: Gambling first aid actions

This section contains statements about what the first aider should do to support someone with gambling problems.

Please rate how important (from 'essential' to 'should not be included') you think it is that each statement be included in the guidelines.

If the person does not want to change their gambling behaviours

***8. The first aider should be aware that the person can only be helped if they are ready to change their gambling behaviours.**

Essential	Important	Don't know/Depends	Unimportant	Should not be included
○	○	○	○	○

***9. If the person does not want to change their gambling behaviours, the first aider should sensitively ask the person if gambling and its consequences are getting in the way of them living the life they want to live.**

Essential	Important	Don't know/Depends	Unimportant	Should not be included
○	○	○	○	○

***10. The first aider should tell the person that they will be available to help the person when they are ready to change their gambling behaviours.**

Essential	Important	Don't know/Depends	Unimportant	Should not be included
○	○	○	○	○

Section 4: Gambling first aid actions (cont.)

This section contains statements about what the first aider should do to support someone with gambling problems.

Please rate how important (from ‘essential’ to ‘should not be included’) you think it is that each statement be included in the guidelines.

Reducing the negative impact of gambling

*11. If the person decides to continue gambling, the first aider should encourage them to reduce the negative impact of gambling by:

	Essential	Important	Don't know/Depends	Unimportant	Should not be included
1. Keeping a photo or other reminder of something they value, but that they are at risk of losing due to gambling (e.g. family, house), in their wallet.	◯	◯	◯	◯	◯

Section 4: Gambling first aid actions (cont.)

This section contains statements about what the first aider should do to support someone with gambling problems.

Please rate how important (from ‘essential’ to ‘should not be included’) you think it is that each statement be included in the guidelines.

Financial strategies

*12. The first aider should know that if they choose to lend the person money, it is unlikely to be repaid. (New)

Essential	Important	Don't know/Depends	Unimportant	Should not be included
◯	◯	◯	◯	◯

Section 4: Gambling first aid actions (cont.)

This section contains statements about what the first aider should do to support someone with gambling problems.

Please rate how important (from ‘essential’ to ‘should not be included’) you think it is that each statement be included in the guidelines.

When there are concerns for safety

*13. The first aider should act to protect family members who may be at risk due to the person's gambling activities.

Essential	Important	Don't know/Depends	Unimportant	Should not be included
◯	◯	◯	◯	◯

Thank you!

Thank you for your time!

That is the end of the Round 3 survey. Thank you very much for your contribution.

If participating in this survey has caused you distress and you wish to talk to someone about this, you can contact any of the following:

Australia: Lifeline on 13 11 14

New Zealand: Lifeline Aotearoa on 0800 543 354

UK: Samaritans on 08457 909090

Republic of Ireland: Samaritans on 116 123

USA: National Suicide prevention Lifeline on 1800 273 TALK (8255)

Canada: National Suicide prevention Lifeline on 1800 273 TALK (8255)

By pressing the *'Next'* button your responses will be registered with our survey software. Once all panel members have lodged their responses, we will collate the data and send you a draft of the guidelines to review.

Kind Regards,

The Mental Health First Aid Research Team
